# Integrated metabolomics, PacBio SMRT, and Illumina sequencing analyses provide insights into molecular profiles associated with radial trunk growth in Chinese fir (*Cunninghamia lanceolata*)

**DOI:** 10.3389/fpls.2026.1739347

**Published:** 2026-04-16

**Authors:** Yating Wen, Quanxu Deng, Xingzhen Mo, Dapei Li, Zengfu Xu, Kaiyong Huang, Kuipeng Li

**Affiliations:** 1 Guangxi Key Laboratory of Forest Ecology and Conservation, Key Laboratory of National Forestry and Grassland Administration on Cultivation of Fast-Growing Timber in Central South China, School of Forestry, Guangxi University, Nanning, China; 2 Guangxi Key Laboratory of Superior Timber Trees Resource Cultivation, key Laboratory of National Forestry and Grassland Administration on Cultivation of Fast-Growing Timber in Central South China, Guangxi Forestry Research Institute, Nanning, China

**Keywords:** Chinese fir, illumina sequencing, metabolome, PacBio SMRT, radial growth, xylem formation

## Abstract

Chinese fir (*Cunninghamia lanceolata*) is a coniferous timber species endemic to China, widely used in furniture manufacturing and construction industries. Metabolite transport and conversion within tree trunk tissues play a crucial role in radial growth. Current research on metabolite changes between the phloem and xylem of tree trunks remains scarce. Owing to the lack of whole-genome sequence information for Chinese fir, studies on the molecular mechanisms underlying its key traits have progressed slowly. This study employed an integrated approach combining metabolomics, PacBio SMRT and illumina sequencing analyze metabolites and transcripts in four distinct regions of Chinese fir: phloem, outer sapwood, inner sapwood, and the transition zone. A total of 398 metabolites were identified in the trunk tissues of Chinese fir. The phloem was found to be primarily enriched in primary metabolites, such as amino acids, sugars, and organic acids, In contrast, the xylem not only accumulated primary metabolites but also exhibited significant enrichment of secondary metabolites including polyphenols, hormones, and pharmacologically active compounds. Among secondary metabolites, the highest number of differentially expressed metabolites were enriched in the phenylpropanoid biosynthesis pathway. Coniferin—a key precursor in lignin biosynthesis, exhibited the highest accumulation among the detected phenolic compounds across all four regions. Among KEGG pathways related to amino acid metabolism, the Arginine and proline metabolism pathway had the highest enrichment of differential metabolites. Metabolites such as glutamate, ornithine, and 4-aminobutanoate in this pathway showed the highest content in the phloem, with a gradual decrease from the phloem to the transition zone. Genes belonging to transcription factor families such as WRKY, bHLH, HB-HD-ZIP, and AP2 were identified, suggesting that these genes may be involved in regulating be involved in regulating xylem formation in Chinese fir. This study provides a foundational molecular profile of metabolic activities in the trunk tissues of Chinese fir and offers valuable information for future research on the molecular mechanisms underlying radial trunk growth in forest trees.

## 1 Introduction

Trees are autotrophic plants, with mature leaves functioning as their primary source organs. Through photosynthesis, they absorb carbon dioxide (CO_2_)and utilize light energy and water to fix carbon, thereby producing carbohydrates (Xu, 2019b). A portion of the carbohydrates fixed via photosynthesis is directly used for cellular metabolism via pathways such as glycolysis, while another portion is stored as sucrose or starch in vacuoles and chloroplasts. However, most of photosynthetically fixed carbohydrates are transported out of mesophyll cells in the form of sucrose and enter the phloem (Zeeman et al., 2010; Pal et al., 2013; Villar-Salvador et al., 2015). The plant root system acts as the primary organ for absorbing water and mineral nutrients, and it also functions as a critical biosynthetic center (Aloni et al., 2004). Roots synthesize cytokinins and secrete root exudates which consist of organic acids, alcohols, sugars, phenols, and phytohormones (Aloni et al., 2004, Aloni et al., 2005; Baetz and Martinoia, 2014; Zhang et al., 2014). Water, nutrients absorbed by roots, and synthesized cytokinins are primarily transported upward through the xylem to promote shoot development and growth (Aloni et al., 2005; Ko et al., 2014; Zhang et al., 2014; Jin et al., 2016).

Plant organs are interconnected and function coordinately through a longitudinal long-distance transport system composed of the phloem and xylem (Aubry et al., 2019). The phloem enables bidirectional transport of organic compounds, such as sugars and amino acids. It loads photosynthates like sugars produced via photosynthesis in mature leaves via the source-end phloem, transports them over long distances, and delivers them to various sink organs (young leaves, roots, flowers, fruits, etc.) to support plant metabolism and growth (Pal et al., 2013; Villar-Salvador et al., 2015). The xylem primarily transports water and mineral ions absorbed by the roots, along with small quantities of organic molecules such as amino acids, iron chelates, and hormones, from the roots upward to aboveground tissues (Dinant and Lemoine, 2010; Pfautsch et al., 2015). Both the phloem and xylem deliver these substances to heterotrophic organs, providing essential nutrients for their growth and development—thus playing a particularly crucial role in the life processes of perennial plants (Pfautsch et al., 2015).

The bark region of the trunk—dominated by the phloem—and the xylem tissue are connected by radially arranged, interconnected parenchyma cells (referred to as rays), which facilitate short-distance lateral transport (Pfautsch et al., 2015). The presence of ray tissue enables bidirectional movement of water, inorganic ions, sugars, amino acids, and phytohormones between these two major vascular tissues. The phloem not only serves as a conduit for distributing photosynthates but also plays a central role in transmitting nutritional status signals, initiating defense responses, and regulating overall plant development (Dinant and Lemoine, 2010). Metabolite accumulation in both the phloem and xylem exhibits both conserved characteristics across tree species and species-specific traits (Turtola et al., 2002; Halder et al., 2021; Shang et al., 2024; Ma et al., 2025b). Secondary metabolic biosynthesis in tree trunks primarily occurs in vascular cambium region, developing xylem, and during heartwood formation. The transition from sapwood to heartwood is accompanied by extensive synthesis and deposition of secondary metabolites, including defensive compounds, such as flavonoids, terpenoids, and lignin. These metabolites form a chemical barrier against biotic stresses while also modulating wood properties and durability (Ma et al., 2021; Jia et al., 2024; Wen et al., 2024).

Tree radial growth refers to the process of trunk lateral thickening which is driven by the differentiation of the vascular cambium (Plomion et al., 2001). Vascular cambium cells undergo proliferation to produce daughter cells, which further differentiate into various cell types through distinct pathways. These daughter cells differentiate outwardly into secondary phloem and inwardly into secondary xylem (i.e., wood tissue). The formation of secondary xylem initiates in the vascular cambium, proceeds through a series of sequential processes cell division and differentiation, cell enlargement, secondary cell wall deposition, and programmed cell death, ultimately undergoing lignification to form mature wood tissue (Mellerowicz and Sundberg, 2008; Zhang et al., 2011). Metabolite translocation and conversion play a crucial role in wood formation, as they enhance energy transport, metabolism, and defense mechanisms (Bergstrom, 2003; Beekwilder et al., 2014; Wang et al., 2016; Conrad et al., 2017; Ma et al., 2021; Li et al., 2023). Radial studies of trees, focused on analyzing metabolite accumulation and differential gene transcription in the phloem and xylem, can advance our understanding of the growth and development pathways in trunk tissues.

Chinese fir(*Cunninghamia lanceolata*)is a coniferous timber species endemic to China, which is widely used in furniture manufacturing and construction industries (Li et al., 2017; Yang et al., 2024). According to data from the Ninth National Forest Inventory released in 2019, the area of cultivated Chinese fir forests in China covers 148 million mu (approximately 9.93 million hectares), with a total stock volume of 7.55×10^8^ m³—ranking first nationwide in both metrics (National Forestry and Grassland Administration, 2019). Due to the lack of whole-genome sequence information for this species, research on the molecular mechanisms underlying key traits in Chinese fir has progressed slowly. PacBio SMRT is a sequencing technology characterized by long read lengths, which enables complete coverage of most transcripts without the need for additional assembly (Gong et al., 2020; Zhou et al., 2022). The translocation and transformation of metabolites within stem tissues play a crucial role in trunk radial growth. In this study, we performed an integrated analysis using third- and second-generation transcriptome sequencing combined with metabolomics to reveal the accumulation patterns of metabolites in the phloem and xylem of Chinese fir and to investigate the association between metabolic activities and gene expression in mature trunk tissues. These findings provide new insights into the metabolic activities occurring in the trunk tissues of Chinese fir and offer valuable information for further research on the molecular mechanisms underlying radial trunk growth in forest trees.

## 2 Materials and methods

### 2.1 Plant material

Chinese fir was planted in Rongshui County, Liuzhou City, Guangxi Zhuang Autonomous Region, China (109° 12′ 51″ E, 25° 12′ 42″ N). Three trees were randomly selected from a 25-year-old Chinese fir stand. Wood cores were collected at breast height (1.3 m) as experimental materials, with three additional trees randomly selected as biological replicates. Immediately after collection, trunk cores were visually separated into four tissue types based on color (PZ: phloem; OSW: outer sapwood; ISW: inner sapwood; TZ: transition zone). The phloem (PZ), the innermost layer of the bark, appeared dark brown. The outer sapwood (OSW), consisting of light-colored wood adjacent to the bark, exhibited high moisture content. The inner sapwood (ISW) was slightly darker in color and had moderately lower moisture content compared to OSW. The transition zone (TZ), located between ISW and the heartwood, was distinguished by a slightly darker boundary. To validate the accuracy of this separation method, total RNA was extracted from each tissue type and assessed by agarose gel electrophoresis. The results revealed a gradual decrease in RNA band intensity from PZ to TZ, which is consistent with the reduced cellular activity and increased programmed cell death characteristic of TZ and ISW tissues. This finding further supports the reliability of our tissue separation approach. Three replicates of each tissue type were extracted from each tree for metabolomic, PacBio SMRT and Illumina Sequencing analysis.

### 2.2 Microstructural analysis

The collected wood cores were separated by location and immediately fixed in 4% paraformaldehyde. After embedding, the samples were sectioned into 12 μm thick transverse sections using a hand-cranked rotary microtome (LEICA). The sections were then stained with a 1% Safranin O-Astra Blue mixture and finally observed under a Leica light microscope.

### 2.3 Metabolite identification and analysis

Tissues (100 mg) were individually ground with liquid nitrogen and the homogenate was resuspended in prechilled 80% methanol, followed by thorough vortexing. The samples were incubated on ice for 5 min and then were centrifuged at 15, 000 × g, 4 °C for 20 min. A portion of the supernatant was diluted with LC-MS grade water to a final methanol concentration of 53%. The samples were subsequently transferred to a fresh Eppendorf tube and then centrifuged again at 15, 000 × g, 4 °C for 20 min. Finally, the supernatant was injected into the LC-MS/MS system [ExionLC™ AD system (SCIEX) coupled with a QTRAP^®^ 6500+ mass spectrometer (SCIEX)] for analysis, which was conducted at Novogene Co., Ltd. (Beijing, China). Samples were injected onto an XSelect HSS T3 column (2.1 × 150 mm, 2.5 μm). In this study, we employed a widely targeted metabolomics approach using a SCIEX QTRAP 6500+ system in multiple reaction monitoring (MRM) mode. Metabolite identification was based on five parameters: parent ion (Q1), daughter ion (Q3), retention time (RT), declustering potential (DP), and collision energy (CE), by matching against Novogene’s in-house database (novoDB). This database was constructed using the same QTRAP 6500+ platform; parameters for some compounds were obtained from authentic standards purchased by Novogene, while others were sourced from shared database information from research institutions, all acquired on the same instrument platform. Therefore, the identification process combined RT matching with MRM transition information, characteristic of a widely targeted strategy rather than untargeted full-scan analysis. The identifications in this study represent a combination of Level 1 and Level 2 confidence. These metabolites were annotated using the KEGG database (http://www.genome.jp/kegg/), HMDB database (http://www.hmdb.ca/) and Lipidmaps database (http://www.lipidmaps.org/). Principal Component Analysis (PCA) and Partial Least Squares Discriminant Analysis(PLS-DA) were performed using metaX, a flexible and comprehensive software for metabolomics data processing. Univariate analysis (t-test) was applied to calculate the statistical significance (P-value). The metabolites with a Variable Importance in Projection (VIP) > 1 and P-value< 0.05, and fold change≥2 or FC ≤0.5were considered differential metabolites.

### 2.4 Construction of PacBio SMRTbell libraries

RNA integrity and potential contamination were assessed via agarose gel electrophoresis. RNA purity was further evaluated using a Nanodrop spectrophotometer (based on OD260/280 ratio), while RNA concentration was accurately quantified using a Qubit fluorometer. The RNA integrity was accurately evaluated using an Agilent 2100 Bioanalyzer. The agarose gel electrophoresis profile (**Supplementary Figure 1**) revealed clear 28S and 18S ribosomal RNA bands in all samples, with the intensity of the 28S band being approximately twice that of the 18S band and no visible smearing, indicating good RNA integrity without significant degradation. A gradual decrease in band intensity was observed from the phloem (PZ) to the transition zone (TZ), which is consistent with the biological characteristics of the transition zone and inner sapwood (ISW) tissues undergoing programmed cell death, reduced metabolic activity, and lower total RNA yield. Nevertheless, the RNA integrity of all samples met the requirements for subsequent transcriptome library construction. The Iso-Seq library was prepared following the Isoform Sequencing (Iso-Seq) protocol using the Clontech SMARTer PCR cDNA Synthesis Kit and the BluePippin Size Selection System protocol as described by Pacific Biosciences (PacBio) protocol (PN: 100-092-800-03). To construct a high-throughput PacBio SMRTbell library, the following procedure was implemented: (1) Enrichment of polyA-containing mRNA using oligo (dT) primers; (2) Reverse transcription of mRNA into cDNA using the SMARTer PCR cDNA Synthesis Kit; (3)Amplification and enrichment of the synthesized cDNA via PCR, with the optimal conditions determined through cycle optimization. (4)Large-scale PCR amplification of size-selected fragments using magnetic beads to obtain sufficient total cDNA. (5)Subjecting the full-length cDNA to damage repair, end repair, and ligation, with SMRTbell adapters to construct the full-length transcriptome library. (6) Digestion with exonuclease to remove cDNA sequences lacking adapters at both ends of the cDNA. (7) Finally, primers and DNA polymerase were bound to form the complete SMRTbell library.

### 2.5 Full-length transcriptome correction

Sequence data were processed using PacBio’s official SMRT Link 7.0 software. yielding subreads sequences. These subreads were then corrected to generate circular consensus sequences (CCS). Sequences were classified as full-length non-chimeric (FLNC) or non-full-length (nFL) based on the presence of 5′-primer, 3′-primer, and poly(A) tail. FLNC were then clustered using the hierarchical n×log(n) algorithm to generate cluster consensus sequences. Finally, these full-length sequences underwent polishing to obtain high-quality consensus sequences for subsequent analyses. The polished consensus sequences were corrected using second-generation sequencing data, followed by redundancy removal using CD-HIT software.

### 2.6 Gene function annotation and non-coding RNA analysis

Gene functional annotation was performed by mapping non-redundant sequences to the following databases: NR (NCBI non-redundant protein sequences); NT (NCBI non-redundant nucleotide sequences); Pfam (Protein family); KOG/COG (Clusters of Orthologous Groups of proteins); Swiss-Prot (A manually annotated and reviewed protein sequence database); KO (KEGG Ortholog database); and GO (Gene Ontology) databases. CDS prediction was conducted using the ANGEL software, and transcript coding potential was predicted using the iTAK software for plant transcription factor prediction, MISA (http://pgrc.ipk-gatersleben.de/misa/misa.html) for SSR identification within the transcriptome, and CNCI, CPC, Pfam-scan, and transcript coding potential was predicted using PLEK. Transcripts predicted to have coding potential were filtered out, while those without coding potential were designated as candidate long non-coding RNAs (lncRNAs). Gene expression levels were estimated for each sample using RSEM, and differential expression analysis between the two conditions/groups was performed using the DESeq R (1.10.1) package. DESeq provide statistical routines for determining differential expression in digital gene expression data using a model based on the negative binomial distribution. The resulting P values were adjusted using the Benjamini and Hochberg’s approach for controlling the false discovery rate. Genes with adjusted P-values < 0.05 identified by DESeq were designated as differentially expressed genes.

### 2.7 Quantitative real-time PCR analysis

Total RNA was extracted using RNAprep Pure Plant Plus Kit(Tiangen, Beijing, China). Reverse transcription was performed using the PrimeScript™ RT Master Mix (TaKaRa, Dalian, China). The primers used were designed using Primer Premier6 software, and their sequences are provided in **Supplementary Table 2**. The qRT-PCR was performed using a TaKaRa real-time quantitative PCR kit (TaKaRa, Dalian, China) on a CFX96 Real-Time System (BIO-RAD, Hercules, CA, USA).The relative gene expression levels were calculated by the ratio = 2 ^-△△Ct^. Three technical replicates were calculated for each sample.

## 3 Results

### 3.1 Metabolite profiles of the phloem, outer sapwood, inner sapwood, and transition zone in Chinese fir

**Figure 1:**
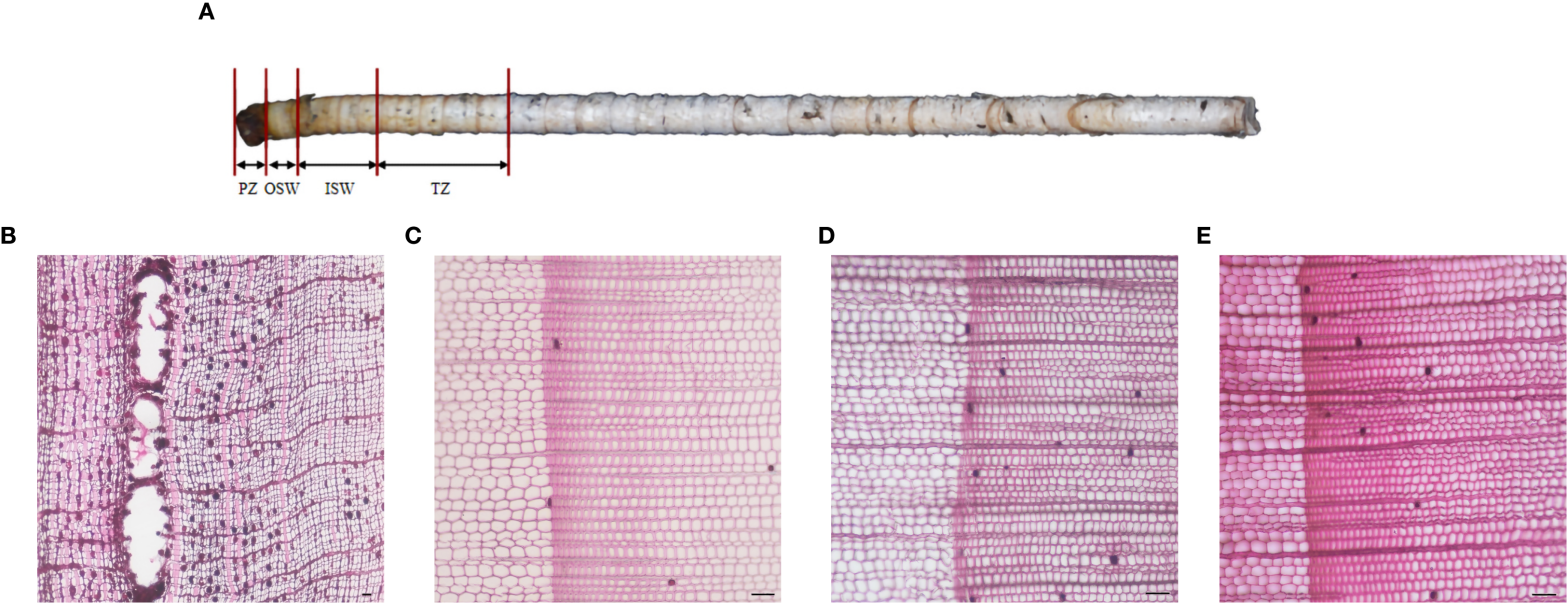
Four different sites of the core of Chinese fir. **(A)** Morphology and appearance of the four tissue sections. **(B)** Microscopic cellular morphology of the phloem. **(C)** Microscopic cellular morphology of the outer sapwood. **(D)** Microscopic cellular morphology of the inner sapwood. **(E)** Microscopic cellular morphology of the transition zone.

**Figure 2:**
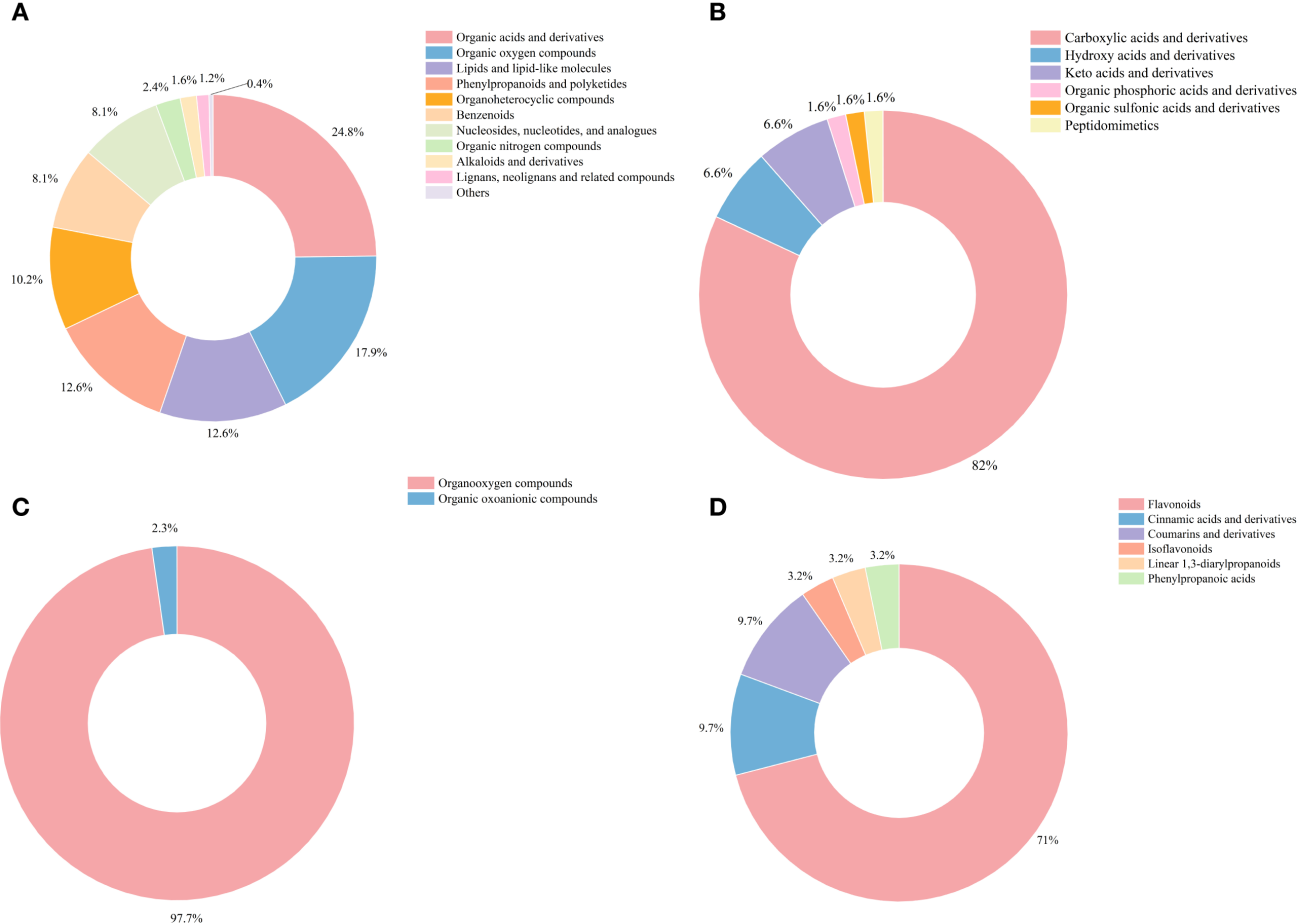
Metabolites from the core of Chinese fir. **(A)** Classification of 398 identified metabolites. **(B)** Categories of Organic acids and derivatives. **(C)** Categories of Organic oxygen compounds. **(D)** Categories of Phenylpropanoids and polyketides.

Wood core of Chinese fir was divided into four radial sections from the outer to the inner layers: phloem (PZ), outer sapwood (OSW), inner sapwood (ISW), and transition zone (TZ). To investigate the radial growth and development process of Chinese fir, metabolomic analysis was performed on these four sections(**Figure 1**). A total of 398 metabolites were detected in this study. Based on the Human Metabolome Database (HMDB), they were grouped into 11 categories: Organic acids and derivatives (24.8%), Organic oxygen compounds (17.9%), Lipids and lipid-like molecules (12.6%), Phenylpropanoids and polyketides (12.6%)Organoheterocyclic compounds (10.2%), Nucleosides, nucleotides, and analogues(8.1%), Benzenoids (8.1%), Organic nitrogen compounds (2.4%), Alkaloids and derivatives (1.6%) Lignans, neo-lignans and related compounds(1.2%), and Others (0.4%)(**Figure 2A**). Within Organic acids and derivatives, six subclasses of substances were detected, including 82% carboxylic acids and derivatives, 6.6% hydroxy acids and derivatives, and 6.6% Keto acids and derivatives (**Figure 2B**). Within Organic oxygen compounds, two subclasses of sub-stances were detected, among which organooxygen compounds accounted for 97.7%(**Figure 2C**). Within the Phenylpropanoids and polyketides category, six subclasses were identified: flavonoids (71%), cinnamic acids and derivatives (9.7%), coumarins and derivatives (9.7%), isoflavonoids (3.2%), linear 1, 3-diarylpropanoids (3.2%), and phenylpropanoic acids (3.2%) (**Figure 2D**).

### 3.2 PCA analysis and systematic clustering

**Figure 3:**
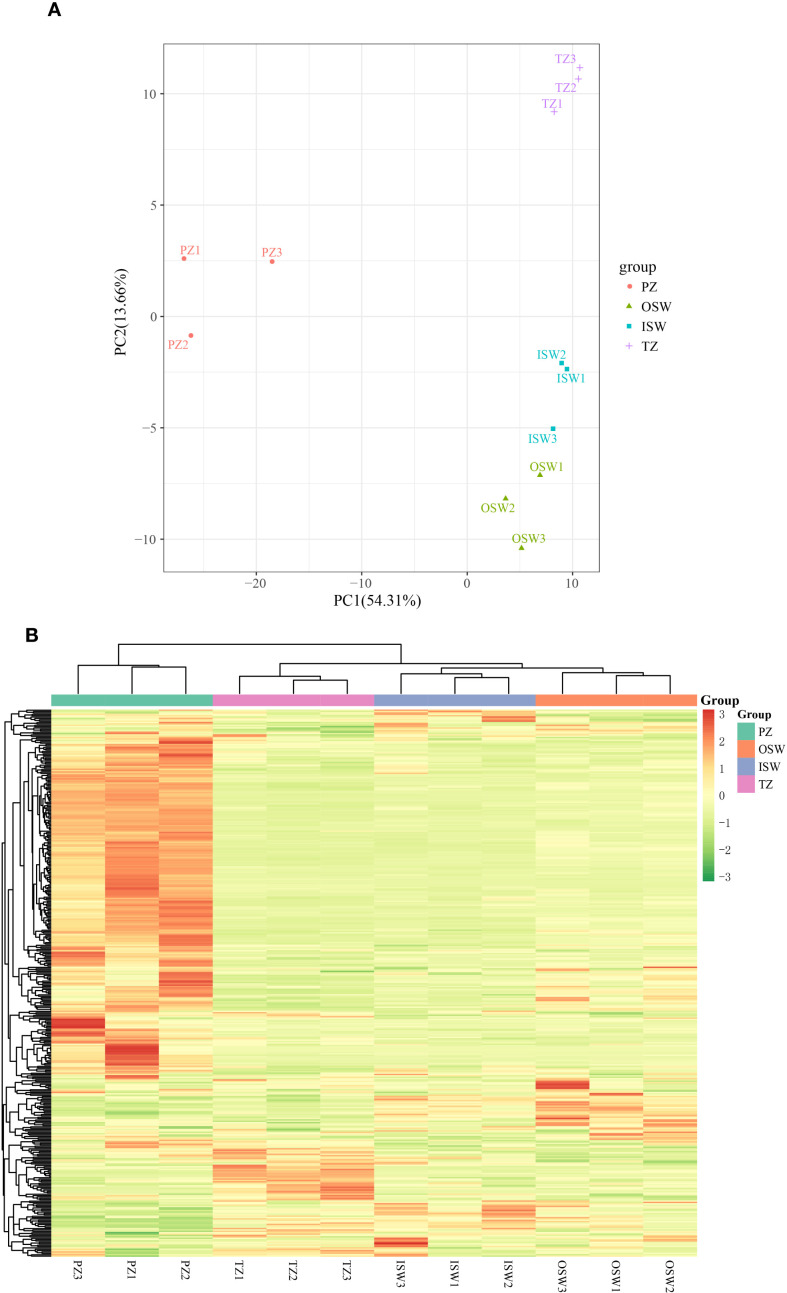
**(A)** Principal component analysis (PCA). **(B)** Heat map visualization. Each metabolite is represented by a row and each sample by a column, with red indicating high abundance and green indicating lower abundance.

In the PCA score plot, the three biological replicates of each tissue were clustered, and all test groups, including the quality control (QC) group, were clearly separate. In the PC1 × PC2 score plot, the first principal component (PC1) explained 54.31% of the total variance. the cumulative variance explained by PC1 and PC2 reached 67.97% (**Figure 3A**). Results indicated that PZ and TZ samples are distinctly separated in the PC1 × PC2 plot, while OSW and ISW samples cluster closely together. This suggests each tissue group hats relatively distinct metabolic profile, with OSW and ISW sharing more similar metabolic characteristics. To further evaluate metabolic differences among the four tissue groups, hierarchical cluster analysis (HCA) was performed on metabolites from each site (**Figure 3B**). The results revealed four distinct clusters which corresponded to the relative differences in metabolite accumulation across the four tissue sections. Most metabolites exhibited higher concentrations in PZ, while fewer metabolites showed elevated levels in OSW, ISW, and TZ.

### 3.3 Differential metabolite analysis in Chinese fir stem tissues

#### 3.3.1 Screening of differential metabolites in different parts of Chinese fir

**Figure 4:**
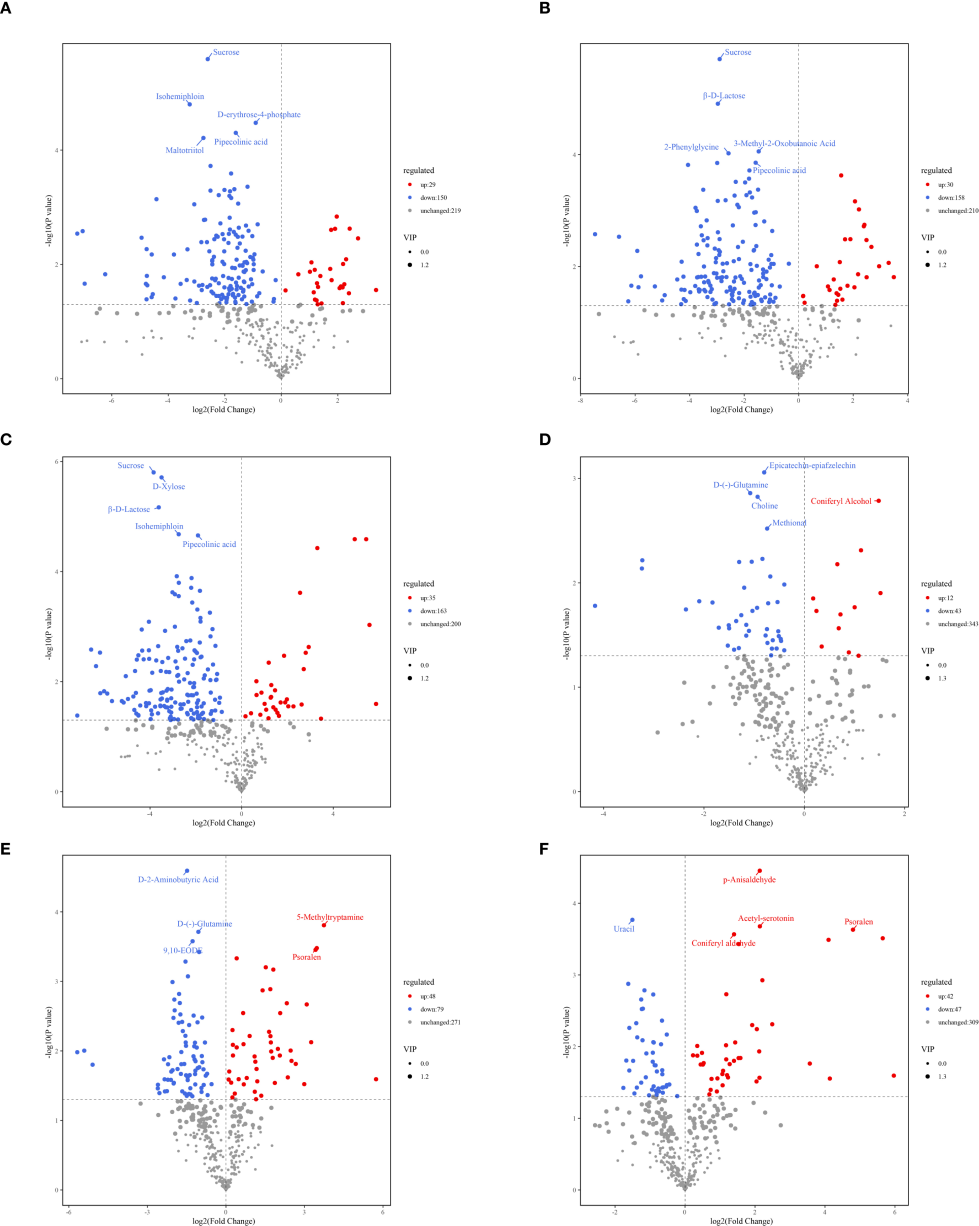
volcano plot of differential metabolites at four sites of Chinese fir SW, TZ, OHW, and IHW. **(A)** PZ *vs*. OSW. **(B)** PZ *vs*. ISW. **(C)** PZ *vs*.TZ. **(D)** OSW *vs*. ISW. **(E)** OSW *vs*.TZ. **(F)** ISW *vs*.TZ.

To further identify differential metabolites among the four tissue regions, metabolites were screened using the criteria of variable importance in projection (VIP) ≥ 1 and fold change (FC) ≥ 2 or FC ≤ 0.5. Volcano plot analysis was conducted to visualize differences in metabolite expression levels among metabolites in different tissue samples. In pairwise comparisons between PZ *vs*. OSW, PZ *vs*. ISW, PZ *vs*. TZ, OSW *vs*. ISW, OSW *vs*. TZ, and ISW *vs*. TZ, the number of differential metabolites was 179 (29 upregulated in OSW), 188 (30 up-regulated in ISW), 198 (35 up-regulated in TZ), 55 (12 up-regulated in ISW), 127 (48 up-regulated in TZ), and 89 (42 up-regulated in TZ) differentially expressed metabolites, respectively (**Figure 4**). The results further confirm significant differences in the relative content of metabolites across the four distinct regions of the Chinese fir wood. Compared with OSW, ISW, and TZ, the PZ region had the highest number of differential metabolites. Fewer differentially expressed metabolites were observed between OSW and ISW. Additionally, the number of differential metabolites between adjacent regions was lower between adjacent regions than between non-adjacent groups, confirming the validity of the sampling strategy.

#### 3.3.2 Differential metabolite analysis of phloem and xylem in Chinese fir

**Figure 5:**
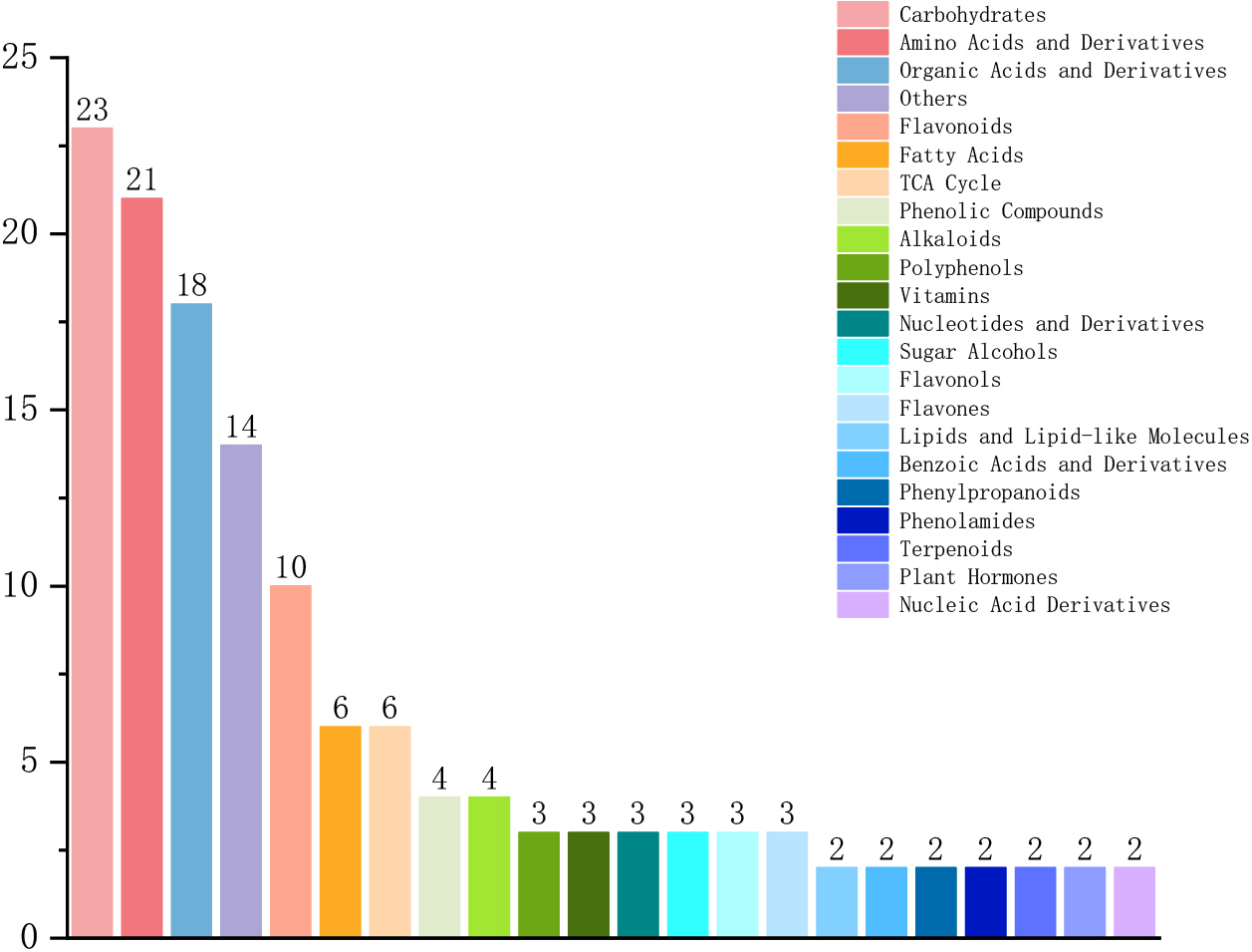
Classification of differential metabolites that specifically accumulate in the phloem, as identified from the common set derived from the PZ *vs*. OSW, PZ *vs*. ISW, and PZ *vs*. TZ comparison groups.

Through pairwise comparisons between the phloem and the other three xylem-related tissues (OSW, ISW, TZ), 138 metabolites were identified that showed consistent differences between PZ and all three xylem tissues. Among these, sugars and amino acids (and their derivatives) ranked first and second in terms of abundance, accounting for 23 (16.7%) and 21 (15.3%) of the total, respectively. This indicates that sugars and amino acids are the primary metabolites in the phloem (**Figure 5**).

#### 3.3.3 Differential metabolite analysis of sapwood and transition zone in Chinese fir

We classified the differentially accumulated metabolites identified from the comparisons among outer sapwood (OSW), inner sapwood (ISW), and transition zone (TZ) based on KEGG pathways. The results revealed that these metabolites were primarily enriched in two pathways: “Amino acid metabolism” (37 metabolites, 22.6%) and “Biosynthesis of other secondary metabolites” (33 metabolites, 20.2%) (**Supplementary Table 1**). Within the “Amino acid metabolism” category, “Arginine and proline metabolism” and “Phenylalanine, tyrosine and tryptophan biosynthesis” contained 5 metabolites each, accounting for 13.5% of the total metabolites in this category. In the “Biosynthesis of other secondary metabolites” category, “Phenylpropanoid biosynthesis” and “Biosynthesis of various plant secondary metabolites” contained 6 metabolites each, representing 18.1% of the total metabolite this category (**Table 1**).

**Table 1:** KEGG classification and quantity of differential metabolites between sapwood and transition zone assigned to Amino acid metabolism and Biosynthesis of other secondary metabolites.

KEGG_Pathway_Level2	KEGG_Pathway_Level3	Number
Amino acid metabolism	**Total**	**37**
	ko00220 Arginine biosynthesis ko00250 Alanine, aspartate and glutamate metabolism ko00260 Glycine, serine and threonine metabolism ko00270 Cysteine and methionine metabolism ko00280 Valine, leucine and isoleucine degradation ko00290 Valine, leucine and isoleucine biosynthesis ko00300 Lysine biosynthesis ko00310 Lysine degradation ko00330 Arginine and proline metabolism ko00340 Histidine metabolism ko00350 Tyrosine metabolism ko00360 Phenylalanine metabolism ko00380 Tryptophan metabolism ko00400 Phenylalanine, tyrosine and tryptophan biosynthesis	1 3 3 3 2 3 1 1 5 1 3 3 3 5
Biosynthesis of other secondary metabolites	**Total**	**33**
	ko00261 Monobactam biosynthesis ko00901 Indole alkaloid biosynthesis ko00940 Phenylpropanoid biosynthesis ko00941 Flavonoid biosynthesis ko00943 Isoflavonoid biosynthesis ko00944 Flavone and flavonol biosynthesis ko00945 Stilbenoid, diarylheptanoid and gingerol biosynthesis ko00950 Isoquinoline alkaloid biosynthesis ko00960 Tropane, piperidine and pyridine alkaloid biosynthesis ko00965 Betalain biosynthesis ko00966 Glucosinolate biosynthesis ko00999 Biosynthesis of various plant secondary metabolites	2 1 6 2 1 3 1 2 3 1 5 6

### 3.4 Overview of the full-length transcriptome of Chinese fir

The full-length transcriptome sequencing generated 42.05 Gb of raw high-quality sequencing reads (Polymerase reads), comprising 5, 952, 250 Polymerase reads. After quality control of the raw data, 40.62 Gb of subreads data was obtained, containing 32, 048, 258 Subreads. Through alignment and correction among subreads, 535, 633 reads with consistent sequences were obtained. By classifying circular consensus sequences (CCSs) based on whether they contained 5’-primer, 3’-primer, or poly(A) tail, 319, 391 FLNC (Full-Length Non-Chimera) sequences were identified. FLNC sequences derived from the same transcript were clustered using the hierarchical n×log(n) algorithm to remove redundancy, yielding cluster consensus sequences. The consensus sequences were polished using Arrow software, resulting in 34, 396 polished consensus reads. To improve sequencing accuracy, transcript sequences were further refined using LoRDEC software with second-generation sequencing data for correction to enhance sequencing accuracy. Finally, CD-HIT software was used for sequence alignment and clustering to eliminate redundancy, resulting in 14, 721 high-quality transcripts. This workflow ensured the integrity of the full-length transcriptome data (**Table 2**).

**Table 2:** Summary of SMRT sequencing data.

Category	0-1kb	1kb-2kb	2kb-3kb	3kb-4kb	Above4kb	Total
Subreads	16077770	10236503	4093762	1157029	483194	32048258
CCS	146549	199382	121423	44225	24054	535633
FLNC	86796	128304	72032	23586	8673	319391
Polished consensus	8875	14204	8216	2466	635	34396

### 3.5 Functional annotation of nonredundant isoforms

**Figure 6:**
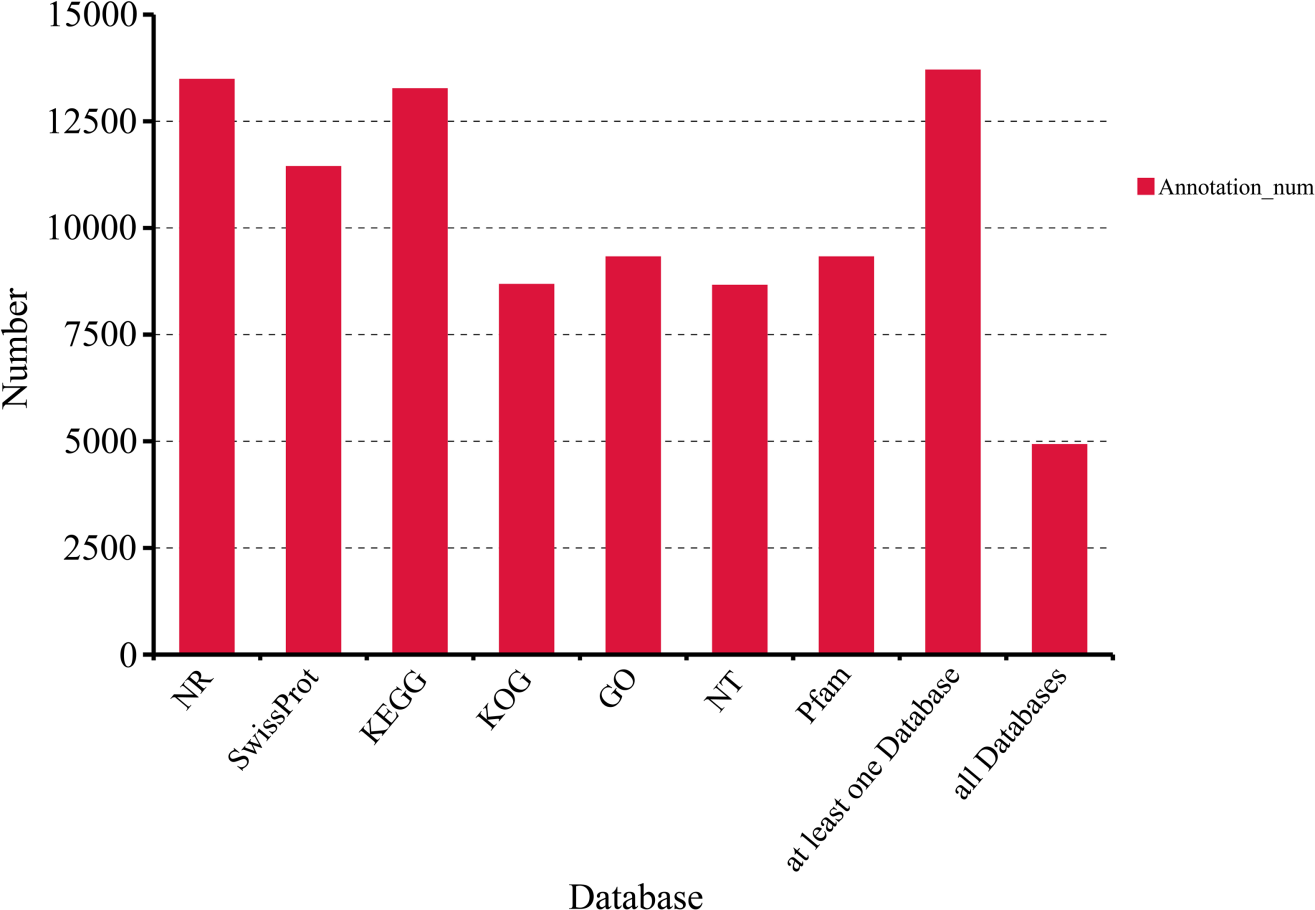
Annotation against the Nr, Nt, Pfam, KOG/COG, Swiss-Prot, KEGG, and GO databases.

Among the non-redundant isoforms, 13, 689 were annotated in at least one of the seven major databases: Nr, NT, Pfam, KOG/COG, Swiss-Prot, KEGG, and GO. A total of 4, 911 isoforms were matched in all seven public databases (**Figure 6**). Among these databases, the Nr database had the highest match rate, annotating 13, 472 matched isoforms. The top seven species with the highest BLAST match scores to Chinese fir isoforms were Picea sitchensis, Amborella trichopoda, Nelumbo nucifera, Marchantia polymorpha, Vitis vinifera, Elaeis guineensis, and Phoenix dactylifera(**Supplementary Figure 2**). This indicates a high degree of homology between Chinese fir and Picea sitchensis, both of which belong to gymnosperms. In the KOG database, 8, 664 isoforms were assigned to 25 functional categories, with the largest being “General function prediction only” (1, 561 isoforms) and the smallest being “Cell motility” (2 isoforms) (**Supplementary Figure 3**). To confirm the functions and pathways of the isoforms, 9, 310 isoforms were subjected to GO annotation. For biological process, the top three subgroups were metabolic process (4, 569 isoforms), cellular process (4, 374), and single-organism process (3, 154). For cellular component, the top three subgroups were cell (2, 027), cell part (2, 027), and organelle (1, 504). For molecular function, the top three subgroups were binding (5, 575), catalytic activity (4, 245), and transporter activity (400) (**Supplementary Figure 4**). In the KEGG classification, 13, 252 isoforms were assigned to six major categories (Metabolism, Human Diseases, Environmental Information Processing, Cellular Processes, Genetic Information Processing, Organismal Systems). Among these categories, the largest number of isoforms was found in “Translation” (695) within the Genetic Information Processing branch, followed by “Signal transduction” (662) in the Environmental Information Processing branch (**Supplementary Figure 5**).

**Figure 7:**
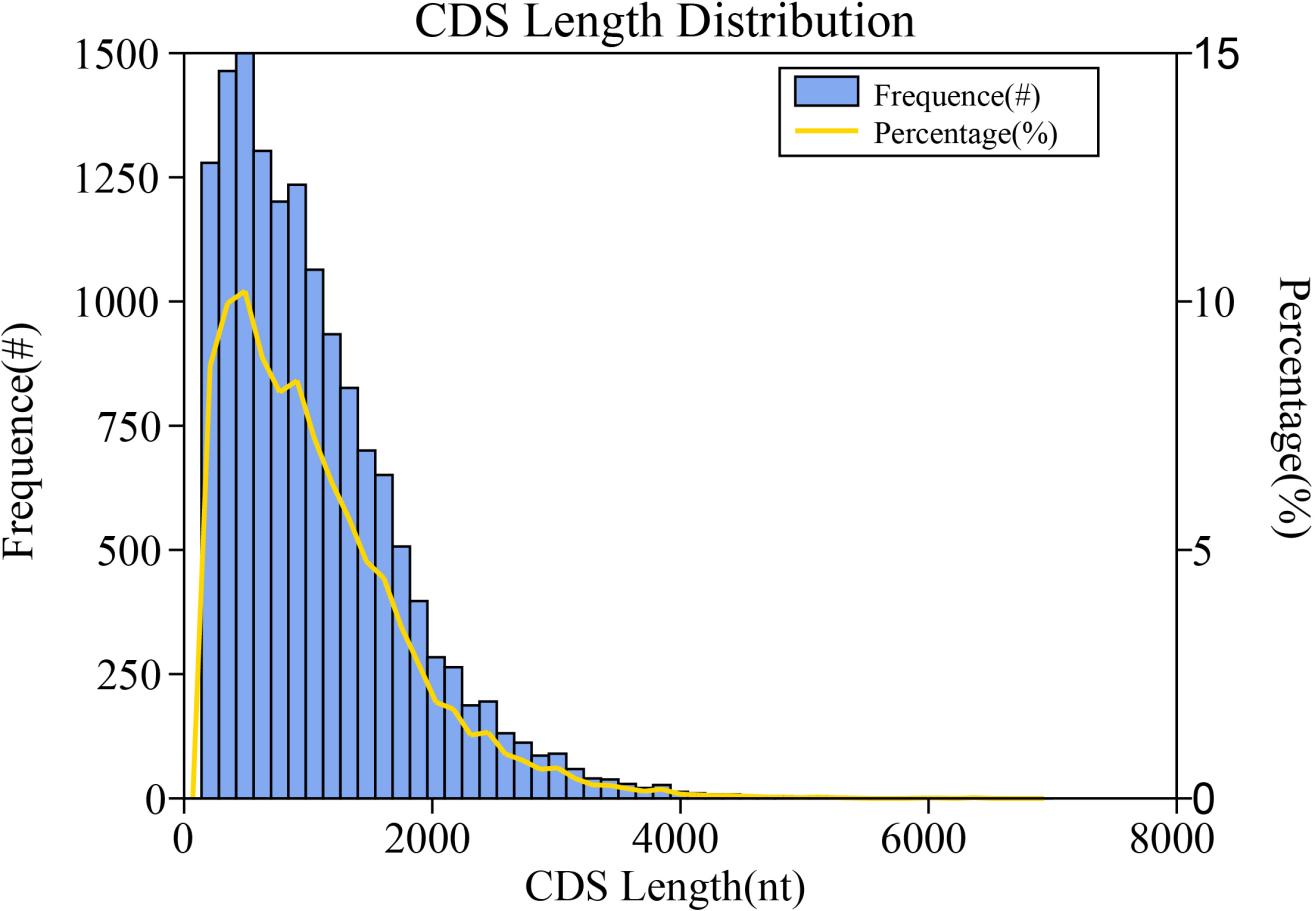
CDS dength distribution. X-axis: Length (nt), Y-axis: Frequency (Reads).

In the full-length transcriptome sequencing results, predicting protein-coding regions facilitates preliminary gene function analysis and serves as the foundation for subsequent protein structural analysis. In this study, a total of 14, 690 CDS were predicted, with lengths ranging from 49nt to 2, 131nt (**Figure 7**). The majority of CDS lengths were concentrated within the 50–700 nt range. Additionally, iTAK software was used for plant transcription factor prediction, identifying 680 TFs distributed across 59 families. The C3H family contained 45 members, followed by bZIP (41), bHLH (40), and Trihelix (38) (**Supplementary Figure 6**). Furthermore, 1, 032 transcripts remained unannotated in any of the seven major databases, suggesting the presence of novel non-coding RNAs (ncRNAs) in Chinese fir. We performed coding potential prediction on isoforms obtained after CD-HIT deduplication using CNCI, CPC2, Pfam-scan, and PLEK. This process ultimately identified lncRNAs—non-protein-coding RNA molecules with lengths exceeding 200 nt. Among all non-redundant isoforms, 1, 161 lncRNAs were identified that were consistently detected by all four prediction methods, ranging in length from 205 to 6, 332 nt(**Supplementary Figure 7**).

### 3.6 Differential gene and transcription factor analysis

**Figure 8:**
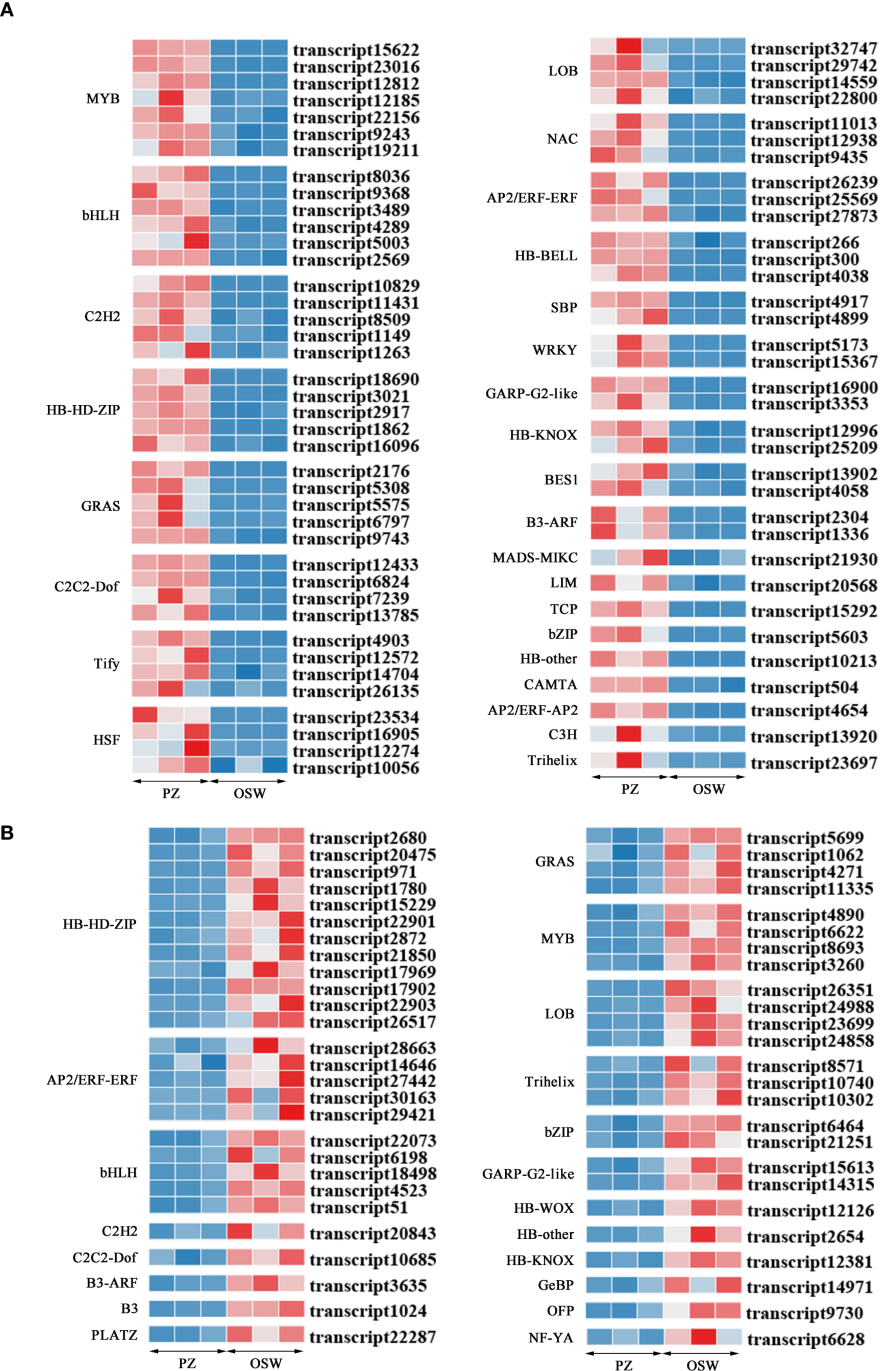
Differentially expressed transcription factors in PZ and OSW. Identified with thresholds of p ≤ 0.05 and |log_2_FC| ≥ 2. **(A)** Transcription factors upregulated in PZ. **(B)** Transcription factors upregulated in OSW.

Pairwise transcriptome comparisons between PZ *vs*. OSW, PZ *vs*. ISW, PZ *vs*. TZ, OSW *vs*. ISW, OSW *vs*. TZ, and ISW *vs*. TZ identified 1593, 2091, 1525, 111, 723, and 375 DEGs, respectively. Among these, 782 DEGs were upregulated in PZ (PZ *vs*. OSW), 1150 DEGs were upregulated in PZ (PZ *vs*. ISW), 878 DEGs were upregulated in PZ (PZ *vs*. TZ), 91 DEGs were upregulated in OSW (OSW *vs*. ISW), 357 DEGs were upregulated in OSW(OSW *vs*. TZ), 79 DEGs were upregulated in ISW (ISW *vs*. TZ) (**Table 3**). Comparing PZ and OSW, we identified 133 differentially expressed transcription factors. Compared with OSW, 74 transcription factors were upregulated in PZ, while 59 transcription factors were upregulated in OSW. Transcription factors upregulated in PZ were annotated to the following families: MYB, bHLH, C2H2, HB-HD-ZIP, GRAS, C2C2-Dof, Tify, HSF, LOB, NAC, AP2/ERF-ERF, HB-BELL, SBP, WRKY, GARP-G2-like, HB-KNOX, B3-ARF, BES1, HB-other, AP2/ERF-AP2, TCP, LIM, Trihelix, bZIP, CAMTA, C3H, MADS-MIKC (**Figure 8A**).Among these, the MYB family had 7 members, the bHLH family had 6 members, and the C2H2, HB-HD-ZIP, and GRAS families each had 5 members. Transcription factors upregulated in OSW were annotated to the following families: HB-HD-ZIP, WRKY, bHLH, AP2/ERF-ERF, GRAS, MYB, LOB, Trihelix, bZIP, GARP-G2-like, GeBP, HB-KNOX, NF-YA, C2C2-Dof, B3, OFP, HB-WOX, C2H2, B3-ARF, HB-other, and PLATZ (**Figure 8B**). The HB-HD-ZIP family contained the highest number of transcription factors (12), followed by the WRKY family (7) and the bHLH family (5). Within the HB-HD-ZIP family, transcript2680 exhibited the highest expression level. Among the AP2 family, transcript30163 showed the most significant upregulation in OSW, increasing by 7-fold.

**Table 3:** Summary table of differentially expressed genes across the four trunk tissues of Chinese fir.

Comparison	Total DEGs	Up-regulated	Down-regulated	Key pathways enriched
PZ *vs* OSW	1593	782	811	Linoleic acid metabolism Plant hormone signal transduction
PZ *vs* ISW	2091	1150	941	Phenylpropanoid biosynthesis Linoleic acid metabolism
PZ *vs* TZ	1525	878	647	Diterpenoid biosynthesis Flavonoid biosynthesis
OSW *vs* ISW	111	91	20	Phenylpropanoid biosynthesis Pentose and glucuronate interconversions
OSW *vs* TZ	723	357	366	Diterpenoid biosynthesis Terpenoid backbone biosynthesis
ISW *vs* TZ	375	79	296	Terpenoid backbone biosynthesis Flavonoid biosynthesis

### 3.7 Integrated transcriptome and metabolome analysis of key metabolic pathways

**Figure 9:**
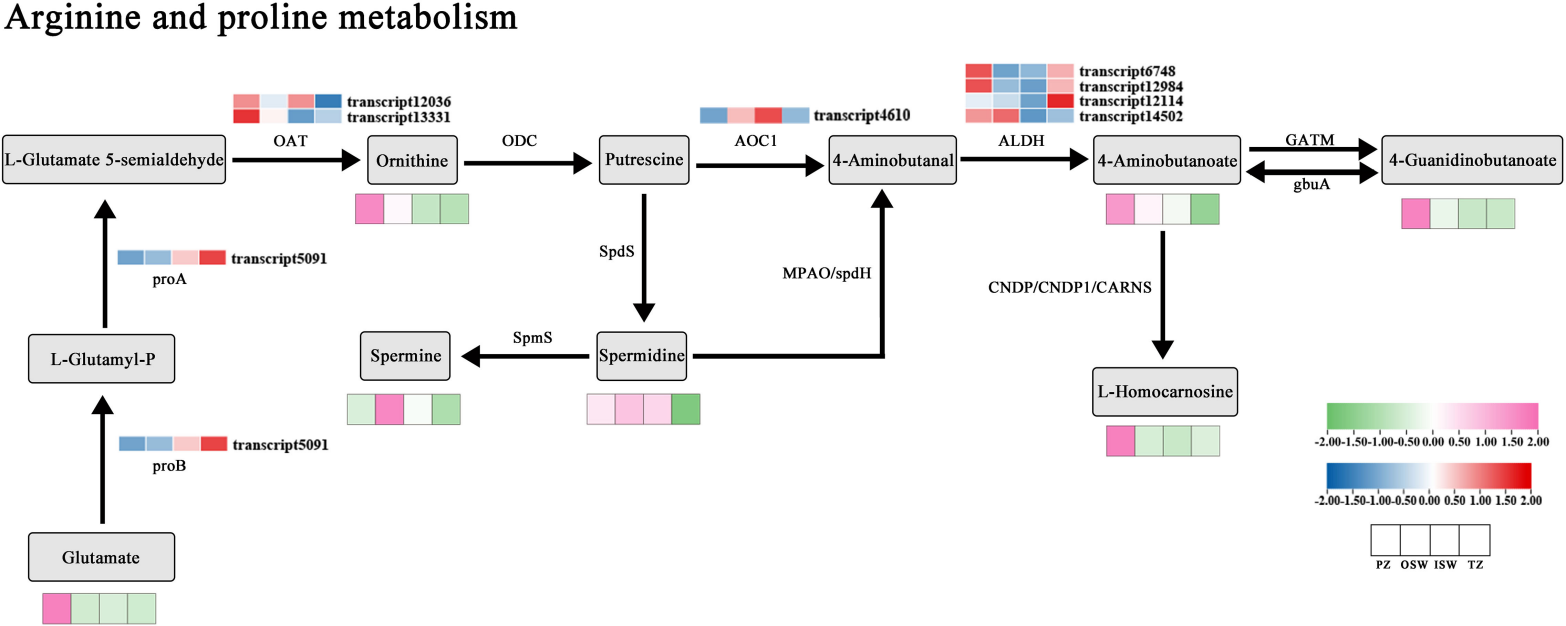
The transcriptional and metabolic landscape of the Arginine and proline metabolism pathway in Chinese fir. A pink-to-green gradient heatmap depicts the relative abundance of metabolites in each tissue section, while a red-to-blue gradient heatmap represents the expression levels of genes in each section.

**Figure 10:**
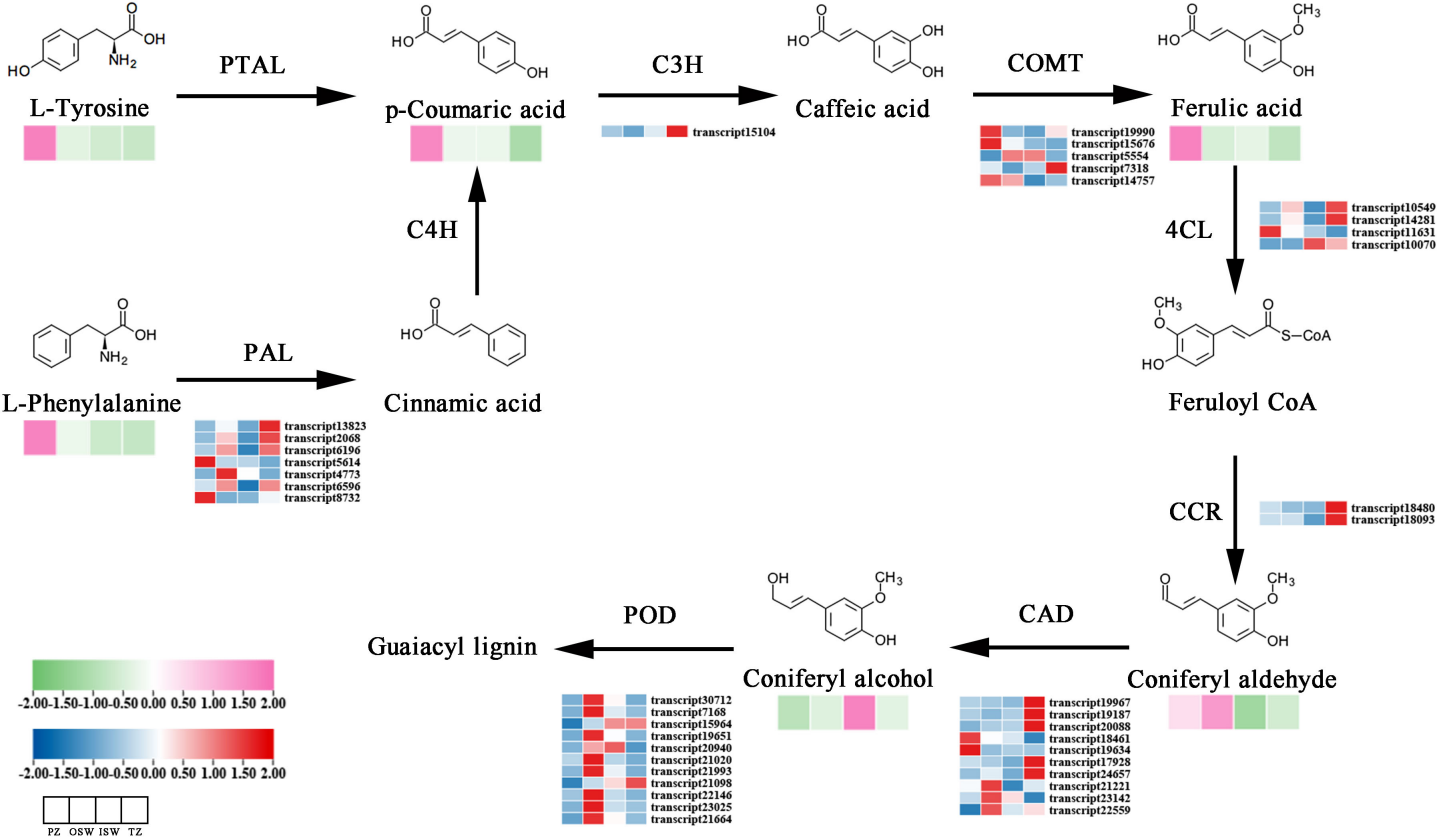
The transcriptional and metabolic landscape of the Phenylpropanoid biosynthesis Pathway in Chinese Fir. A pink-to-green gradient heatmap displays the identities and relative abundance of metabolites in each tissue section, while a red-to-blue gradient heatmap indicates the gene expression levels in each section.

Differentially expressed metabolites (DEMs) identified in the OSW, ISW, and TZ were classified based on the KEGG database. The most abundant pathway associated with these DEMs was Amino acid metabolism, followed by Biosynthesis of other secondary metabolites. Within the “Amino acid metabolism” category, “Arginine and Proline metabolism” was the most prominent subpathway, while “Phenylpropanoid biosynthesis” was the dominant subpathway in “Biosynthesis of other secondary metabolites”. Therefore, we focused on analyzing the Arginine and proline metabolism and Phenylpropanoid biosynthesis pathways. To clarify the relationship between gene expression patterns and metabolite accumulatio, a red-to-blue gradient heatmap was generated to visualize metabolite levels in Arginine and proline metabolism and Phenylpropanoid biosynthesis based on metabolomics and transcriptomics data. We also categorized enzyme-coding genes related to these pathways were extracted from the transcriptome data and created a pink-to-green gradient heatmap based on FPKM values. Finally, a core schematic diagram of Arginine and proline metabolism and Phenylpropanoid biosynthesis was constructed (**Figure 9**; **Figure 10**).

#### 3.7.1 Integrated transcriptomic and metabolomic analysis of the arginine and proline metabolic pathways

Compared with the other three sites, certain genes within OAT and ALDH exhibited higher expression levels in the PZ, and metabolites such as Ornithine, 4-Aminobutanoate, 4-Guanidinobutanoate, and L-Homocarnosine showed the highest accumulation in the PZ. Reduced expression of OAT-encoding genes (transcript12036, transcript13331) in TZ likely contributed to the downregulation of ornithine accumulation in TZ. Putrescine serves as a key precursor in downstream branch pathways, where spermidine synthase (SpdS) catalyzes the synthesis of spermidine, and Spermidine is further converted to Spermine via SpmS. Both Spermidine and Spermine accumulated in the external and internal sapwood regions.

**Figure 11:**
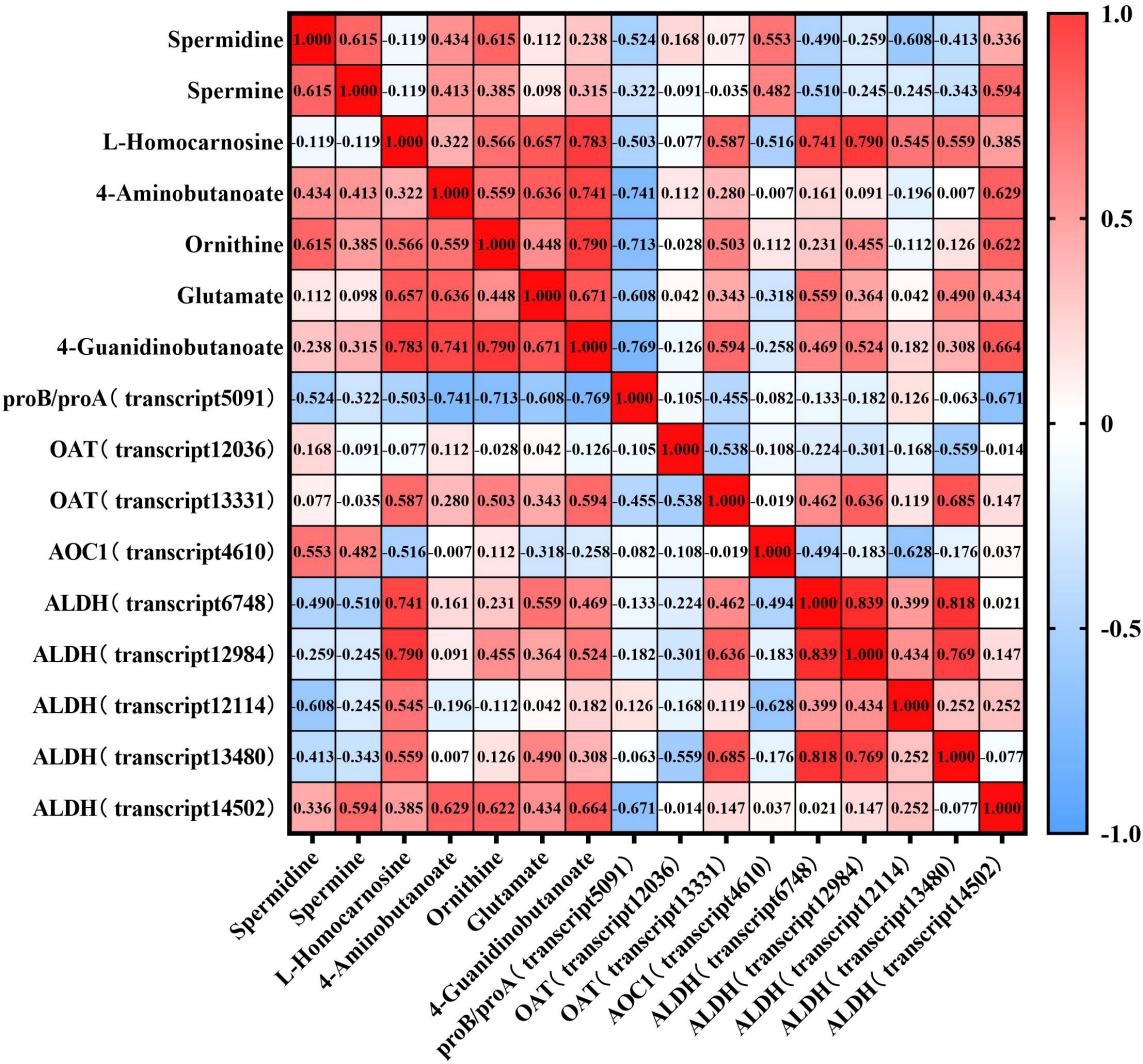
Spearman correlation heatmap of genes and metabolites in the Arginine and Proline Metabolic Pathways. Correlation coefficients (r) were calculated between gene expression (FPKM) and metabolite accumulation. Red indicates positive correlations, blue indicates negative correlations, and color intensity reflects correlation strength.

To further investigate the quantitative relationship between gene expression and metabolite accumulation in the Arginine and Proline Metabolic Pathways, we performed Spearman’s correlation analysis between the FPKM values of key genes in this pathway and the levels of related metabolites (**Figure 11**). ALDH (transcript14502) showed a positive correlation with 4-Aminobutanoate (r = 0.629) and 4-Guanidinobutanoate (r = 0.664), suggesting a potential key role for this gene in the accumulation of these metabolites. ALDH (transcript6748) and ALDH (transcript12984) exhibited strong positive correlations with L-Homocarnosine (r = 0.741 and 0.790, respectively), but showed weak correlations with 4-Aminobutanoate (r = 0.161 and 0.091, respectively). proB/proA (transcript5091) was negatively correlated with all detected metabolites, with relatively strong correlations observed for 4-Aminobutanoate (r = -0.741), Ornithine (r = -0.713), 4-Guanidinobutanoate (r = -0.769), and L-Homocarnosine (r = -0.503), indicating that this gene may act as a negative regulator of this pathway. Spermidine and spermine exhibited generally weak correlations with all detected genes in the pathway (most |r| < 0.5).

#### 3.7.2 Integrated transcriptome and metabolome analysis of the phenylpropanoid biosynthesis pathways

The Phenylpropanoid biosynthesis pathway utilizes L-phenylalanine and L-tyrosine as initial substrates, branching into multiple subpathways that produce to secondary metabolites such as guaiacyl lignin. p-Coumaric acid, which had the highest accumulation in PZ, provides sufficient precursors for downstream pathways. L-Tyrosine, L-Phenylalanine, Ferulic acid, and Coniferin accumulate in the PZ, while Coniferyl aldehyde and Coniferyl alcohol were most abundant in the sapwood (OSW and ISW). In COMT (transcript15676, transcript5554), their expression levels in OSW and ISW were higher than those in TZ, correspondingly, ferulic acid content in OSW and ISW also exceeded that in TZ. Transcript15676 and transcript5554 may represent key regulatory genes in the pathway converting caffeic acid to ferulic acid. Feruloyl CoA provides a key intermediate for lignin synthesis. It is sequentially reduced by CCR and CAD to form coniferyl alcohol, which is ultimately polymerized into guaiacyl lignin by POD. For POD-encoding genes, most genes suggesting that guaiacyl lignin is primarily synthesized in ISW.

**Figure 12:**
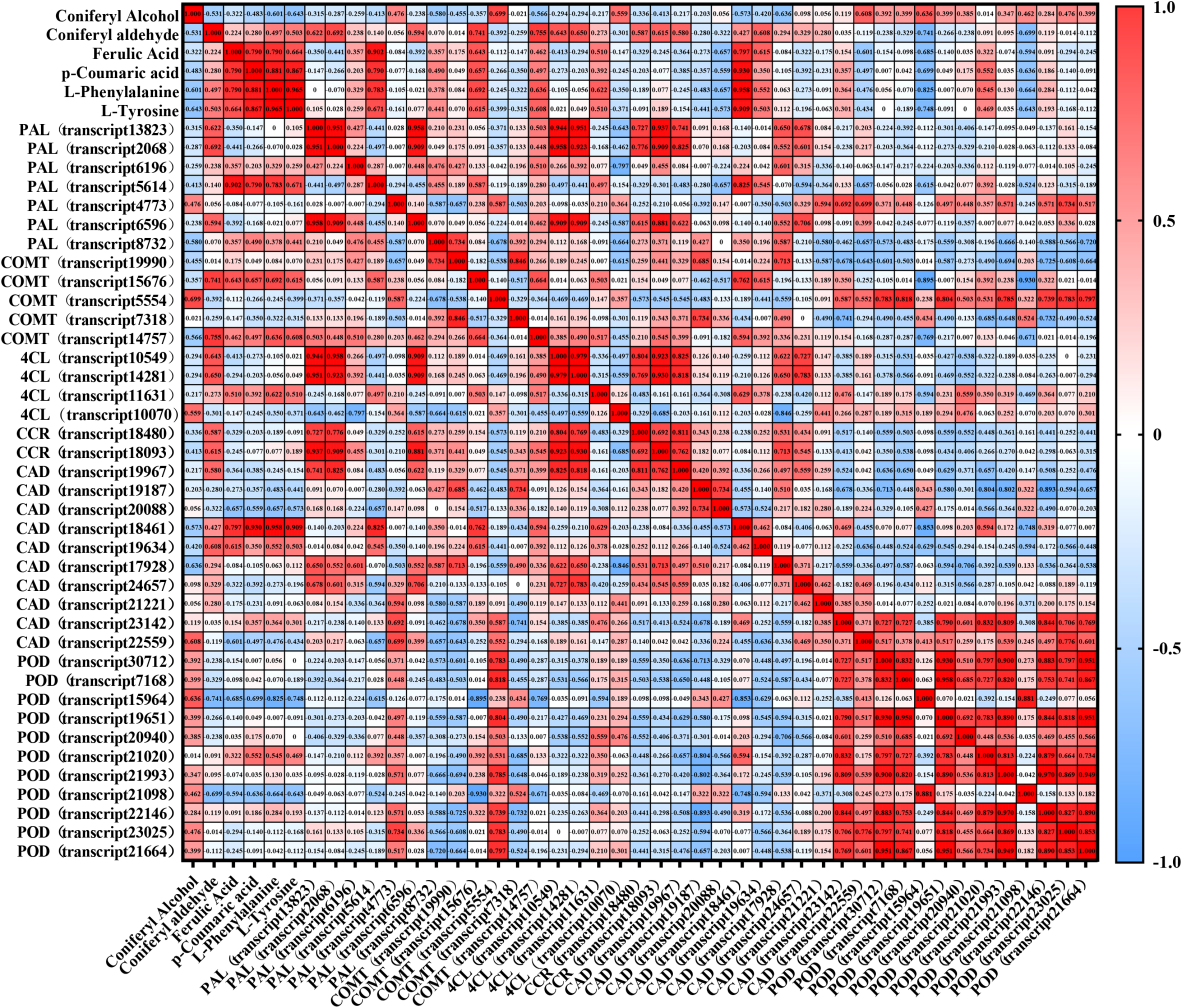
Spearman correlation heatmap of genes and metabolites in the Phenylpropanoid Biosynthetic Pathways. Correlation coefficients (r) were calculated between gene expression (FPKM) and metabolite accumulation. Red indicates positive correlations, blue indicates negative correlations, and color intensity reflects correlation strength.

To further investigate the quantitative relationship between gene expression and metabolite accumulation in the Phenylpropanoid biosynthesis pathway, we performed Spearman’s correlation analysis between the FPKM values of key genes in this pathway and the levels of major metabolites (**Figure 12**). PAL (transcript5614) exhibited strong positive correlations with L-Phenylalanine (r = 0.783), L-Tyrosine (r = 0.671), and Ferulic Acid (r = 0.902). COMT (transcript15676) also showed strong positive correlations with Ferulic Acid (r = 0.643), p-Coumaric acid (r = 0.657), and Coniferyl aldehyde (r = 0.741), further suggesting a key role for transcript15676 in the methylation of caffeic acid to ferulic acid and subsequent lignin biosynthesis. In contrast, COMT (transcript5554) was positively correlated with Coniferyl Alcohol (r = 0.699) but showed a weak correlation with Ferulic Acid (r = -0.112). COMT (transcript19990) was negatively correlated with most metabolites, suggesting a possible role in negative feedback regulation. CCR (transcript18480 and transcript18093) exhibited positive correlations with Coniferyl aldehyde (r = 0.587 and 0.615, respectively) and Coniferyl Alcohol (r = 0.336 and 0.413, respectively), consistent with their role in catalyzing the reduction of aldehydes in the lignin−specific pathway. CAD (transcript19967, transcript19187, and transcript17928) were positively correlated with both Coniferyl aldehyde and Coniferyl Alcohol, with CAD (transcript17928) showing the strongest correlation with Coniferyl Alcohol (r = 0.636), indicating that this gene may play a predominant role in the final steps of monolignol biosynthesis. POD (transcript15964, transcript19651, and transcript20940) were positively correlated with Coniferyl Alcohol (r = 0.636, 0.399, and 0.385, respectively), and several POD genes also exhibited positive correlations with Coniferyl aldehyde (r > 0.5), supporting their function in catalyzing the polymerization of Coniferyl Alcohol into guaiacyl lignin.

### 3.8 Quantitative reverse transcription PCR validation of the transcriptomic data

**Figure 13:**
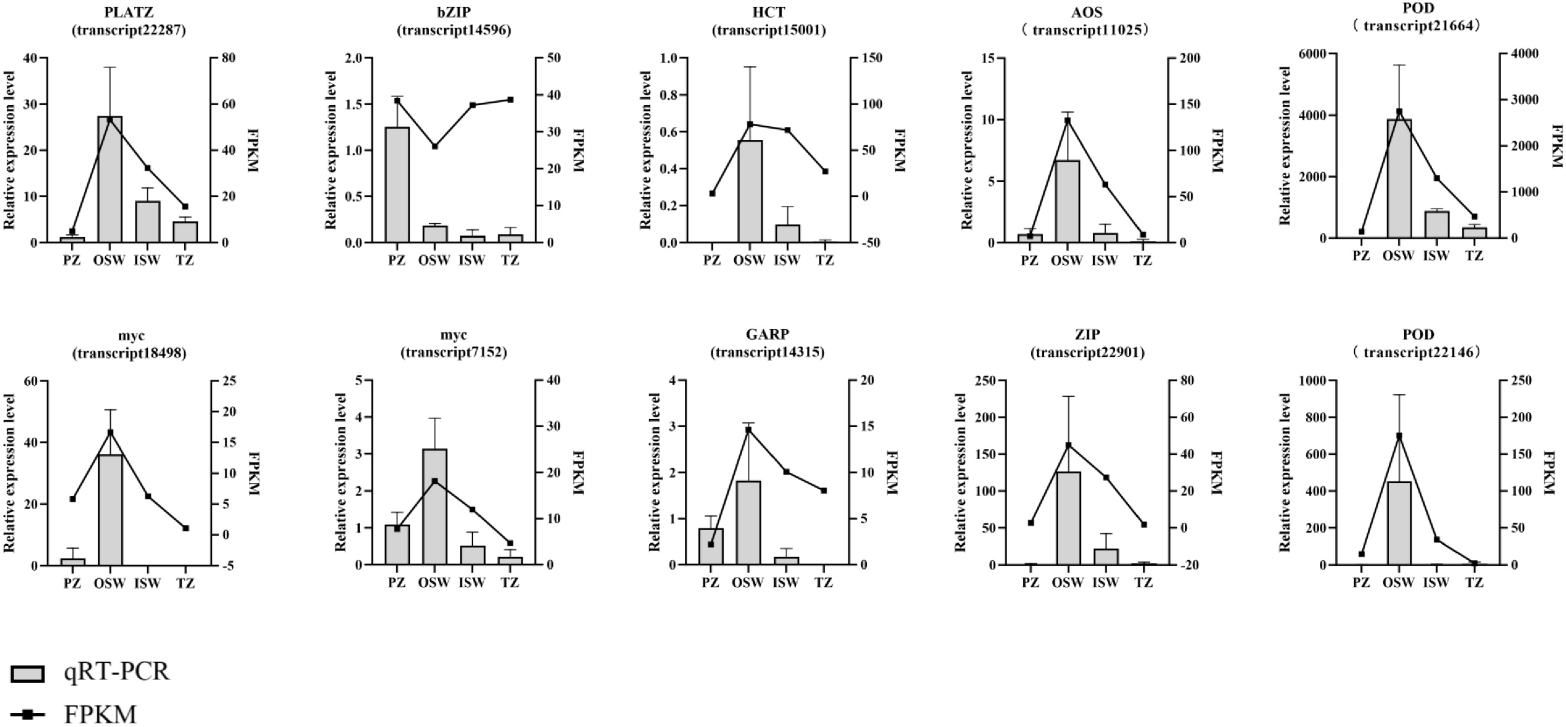
qRT-PCR analysis of expression levels and fragments per million reads (FPKM) of genes in PZ, OSW, ISW and TZ. The bar graphs show the relative expression levels, while the lines show the FPKM values.

To verify the transcriptome data, we selected 10 isoforms for qRT-PCR analysis and examined their relative expression levels in PZ, OSW, ISW and TZ. The results showed general agreement between the FPKM values and qRT-PCR data, indicating that the transcriptome data reliably reflect transcript abundance in this study (**Figure 13**).

## 4 Discussion

The secondary metabolic activities occurring within the trunks and wood of forest trees reflect long-term interactions with the environment and processes that determine wood quality (Rodríguez Anda et al., 2019**;** Li et al., 2020**;** Yang et al., 2021). The secondary metabolites produced during these processes play crucial roles in plant growth and development, stress responses, plant-pathogen interactions, and signal transduction (Li et al., 2020). Thus, elucidating the secondary metabolic processes occurring in tree trunks and wood is essential for cultivating forest resources with high stress tolerance and superior wood quality. This study identified 398 metabolites in the trunk tissues of Chinese fir. These metabolites mainly included primary metabolites such as amino acids and their derivatives, nucleotides and their derivatives, organic acids and their derivatives, and sugars, as well as major secondary metabolites including terpenoids, phenolics, and lignans. Chinese fir belongs to the gymnosperms, an ancient seed plant lineage encompassing conifers, Ginkgo biloba, cycads, and Gnetum (Valderrama-Martín et al., 2024). Extensive research has indicated that typical coniferous woods, such as those from Pinus and Picea species, are characterized by secondary metabolites rich in phenolic and terpenoids compounds (Keeling and Bohlmann, 2006**;** Patyra et al., 2022). The metabolites identified in the trunk tissue of Chinese fir in this study are consistent with the common characteristics of gymnosperm metabolites, yet the enrichment levels of specific metabolites also exhibit species-specific traits. For phenolic compounds, the most abundant ones in Norway spruce (Picea abies) are resveratrol, quercetin, and myricetin(Metsämuuronen and Sirén, 2019). In contrast, coniferin—a key precursor in lignin biosynthesis—showed the highest accumulation in Chinese fir. Its average expression across four sites was 17-fold higher than that of 6-Gingerol, the second most abundant phenolic compound. while 4-Methylcatechol exhibited the lowest expression, only 1.04 × 10⁻^4^ times that of Coniferin. Among terpenoids, β-carotene (a tetraterpenoid) exhibited an average abundance across the four sites 703-fold higher than that of Abietic acid (a diterpenoid resin acid), the second most abundant compound. This contrasts with pine, where monoterpenoid and diterpenoid resin acids dominate (Zulak and Bohlmann, 2010). These differences in metabolite accumulation may explain why Chinese fir wood differs from other gymnosperm woods like pine and spruce in terms of growth rate, wood properties, color, and odor.

Studying radial changes in trees provides Insights into the process from vascular cambium activity to mature wood formation. By analyzing the formation of secondary xylem and phloem, alongside the associated patterns of metabolite transport and conversion, we can uncover the mechanisms governing tree growth and development, secondary cell wall deposition, programmed cell death(PCD) leading to lignification, and the enhancement of energy transport and defense systems. This advances our understanding of the pathways regulating trunk tissue growth and development (Mellerowicz and Sundberg, 2008**;** Zhang et al., 2011**;** Beekwilder et al., 2014**;** Ma et al., 2021**;** Li et al., 2023). Numerous studies have analyzed metabolite migration and transformation pathways during sapwood-to-heartwood formation in xylem (Cao et al., 2020**;** Hu et al., 2022**;** Jia et al., 2024). However, research on metabolite changes between the phloem and xylem remains scarce. In this study, multi-omics technology was employed to jointly analyze metabolite migration and transformation processes in the transition zone from phloem to xylem in Chinese fir. A total of 179 differentially expressed metabolites (DEMs) were identified between PZ and OSW, representing the highest number among the three pairwise comparisons of adjacent regions. Among these DEMs, 150 were upregulated in PZ, primarily consisting of amino acids and their derivatives, sugars, and organic acids and their derivatives. Primary metabolites such as amino acids and sugars are Primary metabolites synthesized in source leaves and transported via the phloem to sink organs (Rentsch et al., 2007**;** Dinant and Lemoine, 2010**;** Tegeder and Masclaux‐Daubresse, 2018**;** Xu, 2019b). No metabolites were found to be exclusively expressed in the phloem in this study. Among the 398 identified metabolites, 118 exhibited a gradual decrease in content from the PZ to the TZ. Plant ray parenchyma cells facilitate short-distance lateral transport of metabolites such as sugars, amino acids, and phytohormones within stem tissues (Pfautsch et al., 2015). We hypothesize that metabolites in the phloem tissue of Chinese fir may undergo radial transport through parenchyma cells to the xylem tissue within the stem. Wood formation in forest trees is regulated by multiple transcription Families. HD-ZIP III transcription families play a crucial role in cambium proliferation and differentiation (Zhu et al., 2013**;** Xu et al., 2019a). NAC and MYB are key regulators of secondary cell wall formation (Demura and Fukuda, 2007**;** Zhong et al., 2010**;** Zhong and Ye, 2014), with the NAC-MYB regulatory network directly targeting structural genes involved in lignin monomers and cellulose biosynthesis, or modulating other transcription factors associated with secondary wall deposition(Demura and Fukuda, 2007). Other transcription factor families, including WRKY, ARF, and bHLH, also participate in wood formation (Xu et al., 2019a**;** Song et al., 2023**;** Deng et al., 2024). The bHLH family regulates lignin monomers biosynthesis (Lu et al., 2013) and controls vascular cell division by forming heterodimers with LHW proteins (Vera-Sirera et al., 2015). In OSW, we detected 61 significantly upregulated TFs, which were annotated as HB-HD-ZIP, WRKY, bHLH, AP2/ERF-ERF, GRAS, MYB, LOB, Trihelix, bZIP, GARP-G2-like, GeBP, HB-KNOX, NF-YA, C2C2-Dof, B3, OFP, HB-WOX, C2H2, B3-ARF, HB-other, and PLATZ. Based on FPKM values exceeding 50 and differential expression ratios greater than 4, we hypothesize that six candidate genes are associated with xylem formation, namely WRKY (Transcript24100, Transcript25281), bHLH (Transcript4523), AP2 (Transcript30163), and HB-HD-ZIP (Transcript17902, Transcript22903).

Trees are a vital component of terrestrial ecosystems, absorbing carbon dioxide (CO_2_) and releasing oxygen (O_2_) through photosynthesis, thereby effectively reducing atmospheric carbon dioxide concentrations. Terrestrial plants sequester over half of the annual global carbon, with the majority stored in wood, making timber a significant carbon sink (Field et al., 1998**;** Luo and Li, 2022). In most tree species, the majority of sucrose produced via photosynthesis is converted into carbon-containing compounds in wood (Turgeon, 1996), serving as the primary form for carbon transport (Rennie and Turgeon, 2009). Within developing wood, and sucrose provides carbon for the synthesis of sugar nucleoside, which act as precursors for cell wall polysaccharide biosynthesis. The phenylpropanoid pathway connects to sugar metabolism through the shikimate pathway (Herrmann and Weaver, 1999). The phenylpropanoid pathway originates from phenylalanine, the end product of the shikimate pathway. Through three sequential reactions, the carbon precursors from phenylalanine are converted to 4-coumaroyl-CoA, which serves as the common precursor for all downstream phenylpropanoid (Xie et al., 2018a). The phenylpropanoid pathway is crucial for plant growth and development, as it produces a diverse range of secondary metabolites including lignin and flavonoid (Cho et al., 2024). Lignin is a key structural component of secondary cell walls and wood (Xie et al., 2018b**;** Ma et al., 2025a). Among the differentially expressed metabolites (DEMs) detected in this study, the highest enrichment was observed in the Phenylpropanoid biosynthesis pathway. L-Tyrosine, L-Phenylalanine, and Ferulic acid exhibited the highest content in the PZ, while Coniferyl aldehyde and Coniferyl alcohol showed peak accumulation in the OSW and ISW, respectively. This indicates that upstream metabolites accumulate in the PZ, whereas downstream metabolites accumulate in the xylem. Transcriptomic analysis detected most enzyme-encoding genes in this pathway, including PAL, C3H, 4CL, CCR, CAD, and POD. These genes were predominantly highly expressed in the OSW or TZ. POD, a peroxidase, is a key redox enzyme in organisms. In plants, POD enzymes are involved in lignin monomer biosynthesis (Barros et al., 2015). Coniferyl alcohol serves as a precursor to lignin monomers, and POD enzymes catalyze its polymerization, ultimately leading to the formation of lignin monomers (Barros et al., 2015**;** Tobimatsu and Schuetz, 2019). The high expression of POD genes in the OSW region, coupled with the low coniferyl alcohol enrichment in this area, suggests that substantial amounts of coniferyl alcohol undergo POD-catalyzed polymerization to form lignin monomers.

Nitrogen is an essential mineral element for plant growth and development. Depending on plant species and environmental conditions, inorganic nitrogen acquired by plants is reduced to amino acids primarily in roots and photosynthetically active source leaves (Rentsch et al., 2007). Chloroplasts and the cytoplasm are the primary sites for the *de novo* biosynthesis of numerous proteinogenic amino acids (Rolin, 2006). After synthesis in source leaves, amino acids are transported through the xylem and phloem to various organs; Thus they accumulate in these vascular tissues and becoming available for uptake by sink tissues and organs such as seeds and new shoots (Tegeder and Masclaux‐Daubresse, 2018). In this study, analysis of KEGG pathways associated with amino acid metabolism revealed that the highest number of differentially expressed metabolites were enriched in the “Arginine and proline metabolism” pathway. The Arginine and proline metabolism pathway is one of the central pathways for the biosynthesis of arginine and proline. These two amino acids can be synthesized from glutamate through a series of enzymatic reactions, and the metabolic flux between arginine, glutamate, and proline is bidirectional (Morris, 2007). Arginine is an essential amino acid for plant growth (Faraji and Sepehri, 2019). Beyond serving as a building block for protein synthesis, it functions as the primary storage and transport form of organic nitrogen in plants. It also acts as a precursor for polyamines and nitric oxide (NO), making it an essential metabolite for numerous cellular processes and developmental events (Jarzyniak and Jasiński, 2014; Ramadan et al., 2019). Proline functions as an osmotic regulator, enhancing plant stress tolerance and reducing cellular damage caused by various abiotic stresses (Jia et al., 2016). Metabolites including Glutamate, Ornithine, 4-Aminobutanoate, 4-Guanidinobutanoate, Spermine, and Spermidine were detected in this pathway. The content of Glutamate, Ornithine, 4-Aminobutanoate, and 4-Guanidinobutanoate was highest in the PZ, gradually decreasing from the PZ to the TZ. Spermine and Spermidine exhibited the highest content in the OSW and the lowest in the TZ. Metabolites such as Glutamate, Spermine, and Spermidine play crucial roles in sustaining cell growth and development within the stem tissues of Chinese fir.

Plant hormones serve as signaling molecules essential signaling molecules for nearly all morphological and physiological processes in plants, including secondary growth (Novák et al., 2017). In this study, five plant hormones five plant hormones or their precursors in Chinese fir: GA7-1, abscisic acid, Methyl indole-3-acetate, salicylic acid, and Salicylic acid O-glucoside were detected. Additionally, eight polyamine compounds were identified, including spermine, spermidine, N-p-Cinnamoylputrescine, N’, N’’-Caffeoyl, feruloylputrescine. Auxins and gibberellins (GAs) synergistically regulate cambial activity, significantly promoting wood growth (Liu et al., 2023). GA stimulates secondary xylem formation of in Arabidopsis and poplar (Ragni et al., 2011**).** Salicylic acid is a key signaling molecule mediating plant immunity and growth; it regulates plant development by modulating cell division and proliferation (Vanacker et al., 2001**;** Fujikura et al., 2020**;** Li et al., 2022). Polyamines are vital growth regulators that exert multi-pathway effects on plant stress responses and tolerance. They positively influence antioxidant mechanisms, enhance the accumulation of osmotic substances, boost photosynthesis, and maintain nitrogen metabolism homeostasis (Choudhary et al., 2012**;** Talaat et al., 2015**;** He et al., 2022**;** Tan et al., 2022). Polyamines interact with all classes of plant hormones, and these interactions enhance plant tolerance to abiotic stresses (Tassoni et al., 2000**;** Napieraj et al., 2023). In the KEGG pathway analysis, most enzyme-encoding genes associated with abscisic acid (ABA) and salicylic acid (SA) synthesis were detected. ABA and SA were primarily enriched in the PZ. Enzyme genes related to ABA synthesis pathways showed high expression mainly in the sapwood, while those associated with SA synthesis pathways exhibited high expression primarily in the TZ. We hypothesize that abscisic acid and salicylic acid can be synthesized within the trunk tissues of Chinese fir, while the other three hormones for which no associated biosynthesis genes were detected in the pathways may not be synthesized during this developmental period. These hormones interact through complex signaling networks, promoting rapid wood growth in Chinese fir while maintaining its adaptability to environmental changes.

In this study, 127 during this developmental period were identified between the TZ and the OSW, while 89 differential metabolites were detected between the ISW and the TZ. Primary metabolites enriched in the xylem transition zone included amino acids and their derivatives, nucleotides and their derivatives, organic acids and their derivatives, fatty acids, etc. In contrast to the secondary metabolites enriched in the phloem, such as flavonoids, alkaloids, flavonols, polyphenols, phenolics, and phenylpropanoids, the transition zone was primarily enriched with secondary metabolites like flavonoids, alkaloids, pharmacologically active compounds, and polyamines. In this study, we detected 11 metabolites with medicinal activity in the woody tissues of Chinese fir. The average abundances of Patchouli alcohol, ANGELOL-K, and 3-dihydrocadambine across the four trunk regions were 4328-fold, 413-fold, and 124-fold higher than that of Poricoic Acid B, which exhibited the lowest average abundance. Alantolactone, 3-dihydrocadambine, ANGELOL-K, Guan-fu base A, and Dihydrodehydrodiconiferyl Alcohol accumulated to the highest levels in OSW. (+)-epipinoresinol-4-O-β-D-glucoside accumulated in ISW, while Poricoic Acid B, Lucidin, and Patchouli alcohol accumulated most in TZ. Patchouli alcohol and Alantolactone exhibit anti-inflammatory and antibacterial activities (Liu et al., 2018**;** Chang et al., 2022). (+)-epipinoresinol-4-O-β-D-glucoside, a lignan component, has been demonstrated to promote cell wall lignification in Eucommia wood. As a secondary metabolite produced in response to environmental stresses and pest/disease infestations, it plays a crucial role in plant growth, development, and stress resistance (Zhang et al., 2022). Its detection in Chinese fir suggests that these compounds may be important in the trees’ defense against biotic and abiotic stresses.

### 4.1 Limitations of the study

Several limitations of this study should be considered when interpreting the results. First, regarding sample size, only three biological replicates (n = 3) were used for each tissue type. Although this sample size is widely accepted in exploratory omics studies and is sufficient to reveal significant and consistent molecular differences among the four distinct trunk tissues of Chinese fir, it should be noted that the conclusions drawn are limited to the specific samples analyzed and are not intended to represent the general characteristics of the entire Chinese fir population. Wood tissues are typically influenced by considerable environmental and genetic variability, and the limited number of replicates may not fully capture the total variation within the population. Therefore, the results of this study should be regarded as a preliminary characterization of the transcriptomic and metabolomic spatial heterogeneity associated with radial growth in Chinese fir, providing a foundation for future validation studies with larger sample sizes and broader sampling sites. Second, in terms of metabolite identification, this study employed a widely targeted metabolomics approach based on Novogene’s in-house database (novoDB). Parameters in this database were derived partly from authentic standards and partly from shared information provided by research institutions. The identifications in this study represent a combination of Level 1 and Level 2 confidence. However, because the database construction did not distinguish the origin of each compound’s parameters, we are unable to assign a specific confidence level to individual metabolites. Nevertheless, through rigorous quality control and multi-parameter matching, the identification results are deemed highly reliable. Third, regarding gene–metabolite associations, this study proposed several speculative conclusions based on correlation analysis (e.g., the regulatory roles of transcription factors). It is important to emphasize that correlation does not imply causation. These inferences provide candidate targets for future functional validation studies, but their true biological functions require experimental verification through approaches such as genetic transformation and enzyme activity assays. Despite these limitations, this study provides an integrated molecular atlas of Chinese fir trunk tissues, offering fundamental data and resources for future research on the metabolic and transcriptional regulation associated with radial growth.

## 5 Conclusions

This study identified a total of 398 metabolites across four distinct regions of Chinese fir. The phloem was primarily enriched primary metabolites such as amino acids, sugars, and organic acids, while the xylem accumulated not only primary metabolites but also secondary metabolites including polyphenols, phytohormones, and pharmacologically active compounds. Among all secondary metabolites, the highest number of differentially expressed metabolites were enriched in the phenylpropanoid biosynthesis pathway. Coniferin—a phenolic compound and key precursor of lignin, exhibited the highest accumulation across all four parts. The Arginine and Proline Metabolism pathway showed the highest enrichment of differential metabolites among KEGG pathways related to amino acid metabolism. Within this pathway, metabolites such as Glutamate, Ornithine, and 4-Aminobutanoate within this pathway exhibited the high-est content in the PZ, with a progressive decrease from the PZ to the TZ. Additionally, we identified six candidate transcription factors potentially involved in regulating the formation of the xylem in Chinese fir. This study enhances our understanding of metabolic activities in Chinese fir trunk tissues and provides a foundation for further exploration of the mechanisms underlying radial growth in tree trunk.

## Data Availability

The raw sequencing data generated in this study have been deposited in the Genome Sequence Archive (GSA) at the National Genomics Data Center, under accession number CRA032184. The data are publicly accessible via the following link: https://ngdc.cncb.ac.cn/gsa.
