# Supplementary material for: Integrated metabolomics, PacBio SMRT, and Illumina sequencing analyses provide insights into molecular profiles associated with radial trunk growth in Chinese fir (Cunninghamia lanceolata)
Source: Front Plant Sci. 2026 Apr 16;17:1739347. doi: 10.3389/fpls.2026.1739347 (PMC13131024; doi:10.3389/fpls.2026.1739347)
Supplement: Supplementary file 1 [file DataSheet1.zip › supplementary files/Supplementary_Material.docx]

Supplementary Material

## Supplementary Figures and Tables

## Supplementary Tables

| **KEGG_Pathway_Level2** | **KEGG_Pathway_Level3** | **Number** |
| --- | --- | --- |
| **Amino acid metabolism** | **Total** | **37** |
|  | ko00220 Arginine biosynthesis  ko00250 Alanine, aspartate and glutamate metabolism  ko00260 Glycine, serine and threonine metabolism  ko00270 Cysteine and methionine metabolism  ko00280 Valine, leucine and isoleucine degradation  ko00290 Valine, leucine and isoleucine biosynthesis  ko00300 Lysine biosynthesis  ko00310 Lysine degradation  ko00330 Arginine and proline metabolism  ko00340 Histidine metabolism  ko00350 Tyrosine metabolism  ko00360 Phenylalanine metabolism  ko00380 Tryptophan metabolism  ko00400 Phenylalanine, tyrosine and tryptophan biosynthesis | 1  3  3  3  2  3  1  1  5  1  3  3  3  5 |
| **Biosynthesis of other secondary metabolites** | **Total** | **33** |
|  | ko00261 Monobactam biosynthesis  ko00901 Indole alkaloid biosynthesis  ko00940 Phenylpropanoid biosynthesis  ko00941 Flavonoid biosynthesis  ko00943 Isoflavonoid biosynthesis  ko00944 Flavone and flavonol biosynthesis  ko00945 Stilbenoid, diarylheptanoid and gingerol biosynthesis  ko00950 Isoquinoline alkaloid biosynthesis  ko00960 Tropane, piperidine and pyridine alkaloid biosynthesis  ko00965 Betalain biosynthesis  ko00966 Glucosinolate biosynthesis  ko00999 Biosynthesis of various plant secondary metabolites | 2  1  6  2  1  3  1  2  3  1  5  6 |
| **Carbohydrate metabolism** | **Total**  ko00010 Glycolysis / Gluconeogenesis  ko00020 Citrate cycle (TCA cycle)  ko00030 Pentose phosphate pathway  ko00040 Pentose and glucuronate interconversions  ko00052 Galactose metabolism  ko00053 Ascorbate and aldarate metabolism  ko00500 Starch and sucrose metabolism  ko00520 Amino sugar and nucleotide sugar metabolism  ko00562 Inositol phosphate metabolism  ko00620 Pyruvate metabolism  ko00630 Glyoxylate and dicarboxylate metabolism  ko00650 Butanoate metabolism | **25**  1  2  1  2  5  2  2  1  1  1  3  2 |
|  | ko00660 C5-Branched dibasic acid metabolism | 2 |
| **Transport and catabolism** | **Total**  ko04148 Efferocytosis | **1**  1 |
| **Energy metabolism** | **Total**  ko00710 Carbon fixation by Calvin cycle | **1**  1 |
| **Folding, sorting and degradation** | **Total**  ko04122 Sulfur relay system | **1**  1 |
| **Lipid metabolism** | **Total**  ko00100 Steroid biosynthesis  ko00564 Glycerophospholipid metabolism  ko01040 Biosynthesis of unsaturated fatty acids | **4**  1  2  1 |
| **Membrane transport** | **Total**  ko02010 ABC transporters | **14**  14 |
| **Metabolism of cofactors and vitamins** | **Total**  ko00130 Ubiquinone and other terpenoid-quinone biosynthesis  ko00670 One carbon pool by folate  ko00730 Thiamine metabolism  ko00760 Nicotinate and nicotinamide metabolism  ko00770 Pantothenate and CoA biosynthesis  ko00780 Biotin metabolism  ko00785 Lipoic acid metabolism | **12**  1  1  3  2  3  1  1 |
| **Metabolism of other amino acids** | **Total**  ko00410 beta-Alanine metabolism  ko00430 Taurine and hypotaurine metabolism  ko00440 Phosphonate and phosphinate metabolism  ko00460 Cyanoamino acid metabolism  ko00470 D-Amino acid metabolism  ko00480 Glutathione metabolism | **12**  3  1  1  4  2  1 |
| **Metabolism of terpenoids and polyketides** | **Total**  ko00900 Terpenoid backbone biosynthesis  ko00904 Diterpenoid biosynthesis  ko00906 Carotenoid biosynthesis  ko00908 Zeatin biosynthesis | **5**  1  1  2  1 |
| **Nucleotide metabolism** | **Total**  ko00230 Purine metabolism  ko00240 Pyrimidine metabolism | **8**  4  4 |
| **Signal transduction** | **Total**  ko04070 Phosphatidylinositol signaling system  ko04075 Plant hormone signal transduction | **3**  1  2 |
| **Translation** | **Total** | **7** |
|  | ko00970 Aminoacyl-tRNA biosynthesis | 7 |

**Table S1.** KEGG classification and quantification of differential metabolites between sapwood and transi-tion zone.

## Supplementary Figures


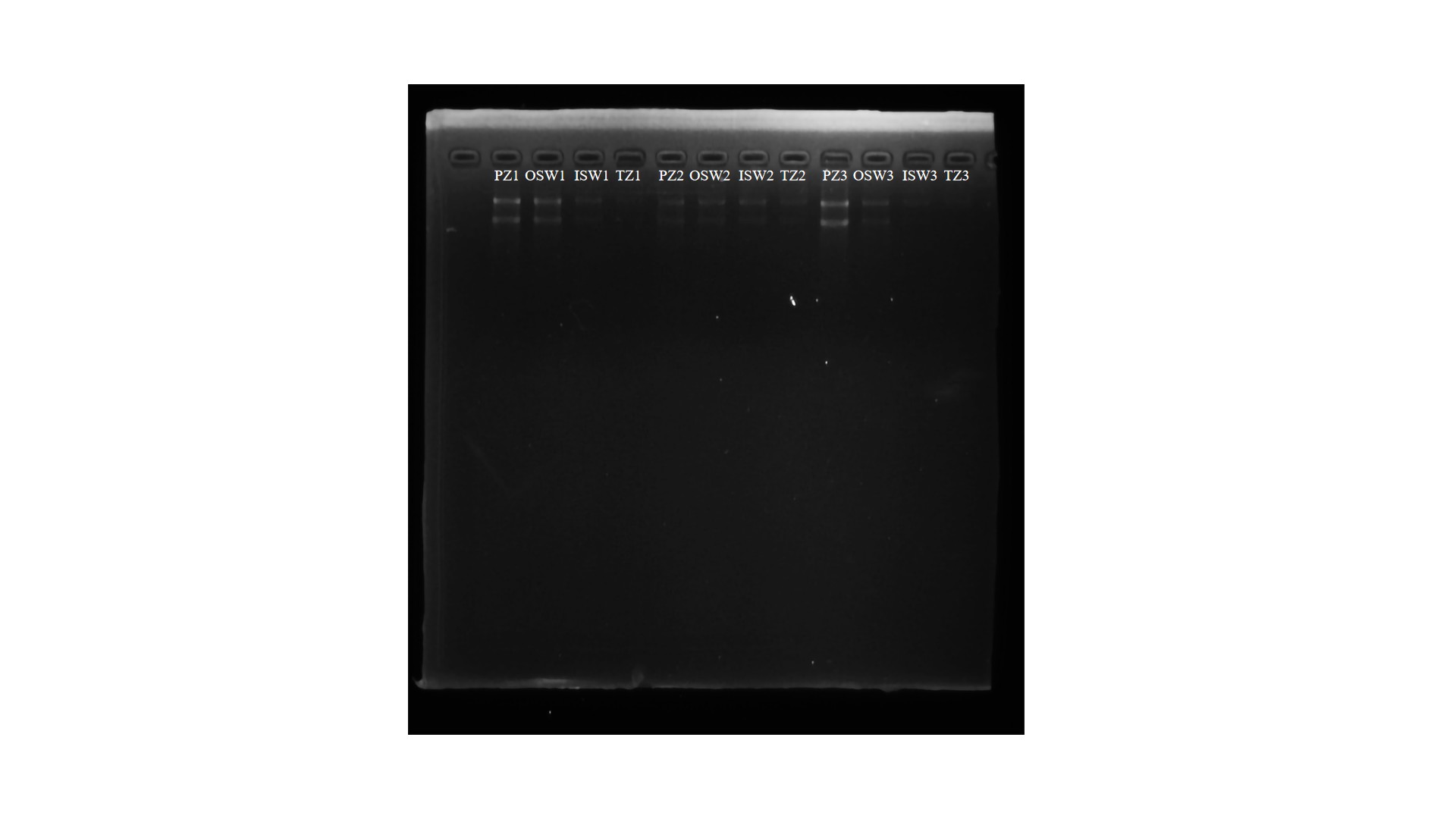


**Supplementary Figure 1.** RNA electrophoretogram of samples from four trunk tissues of Chinese fir.


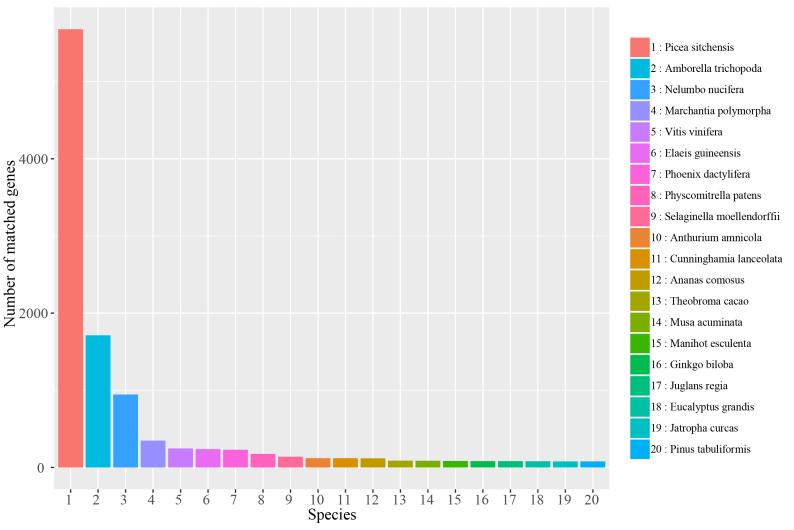


**Supplementary Figure 2.** Species annotation statistics from the NR database.


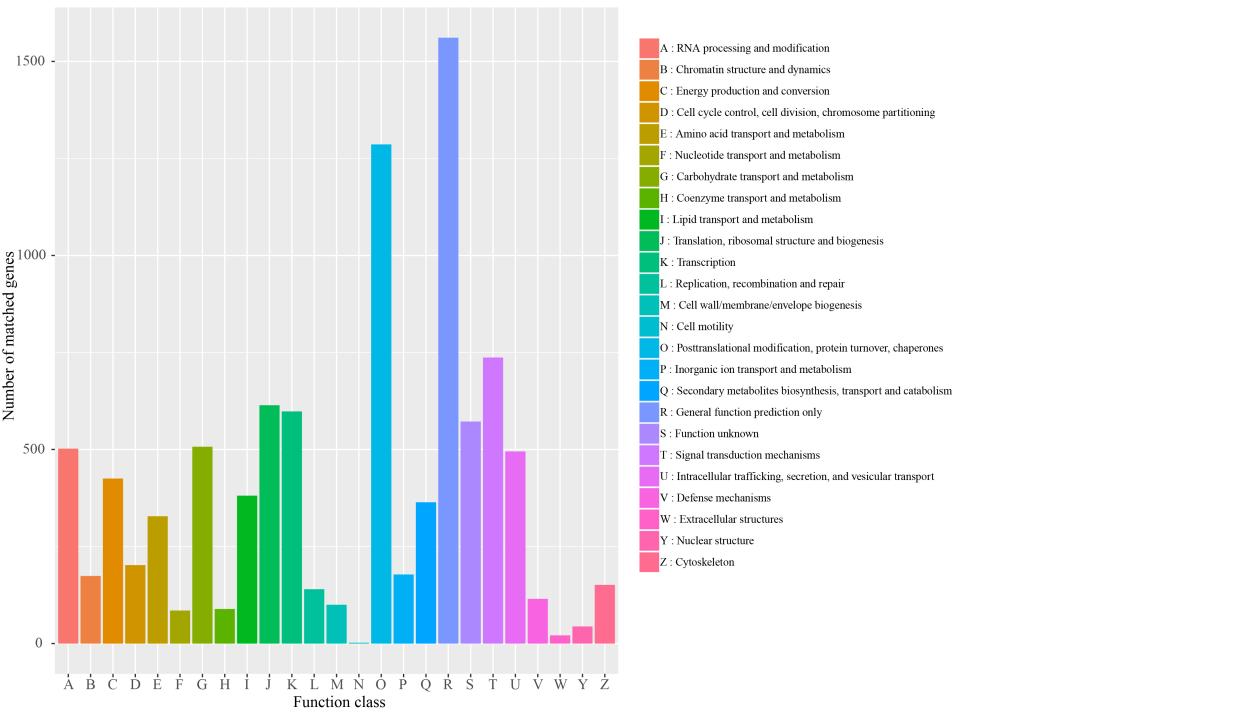


**Supplementary Figure 3.**KOG classification chart.


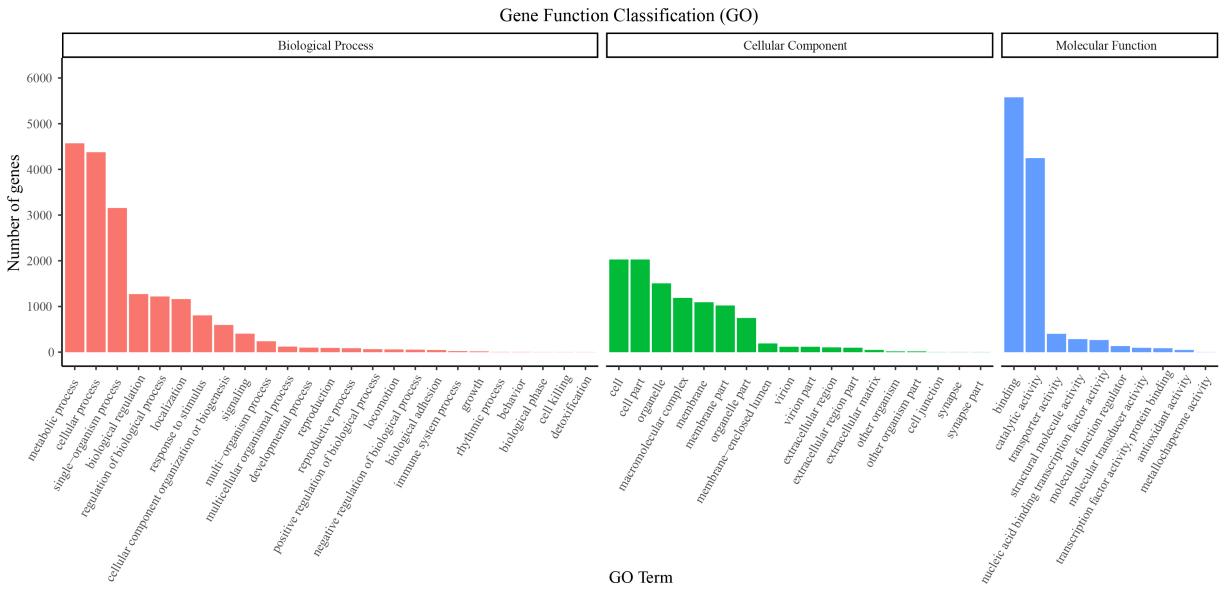


**Supplementary Figure 4.**GO classification chart.


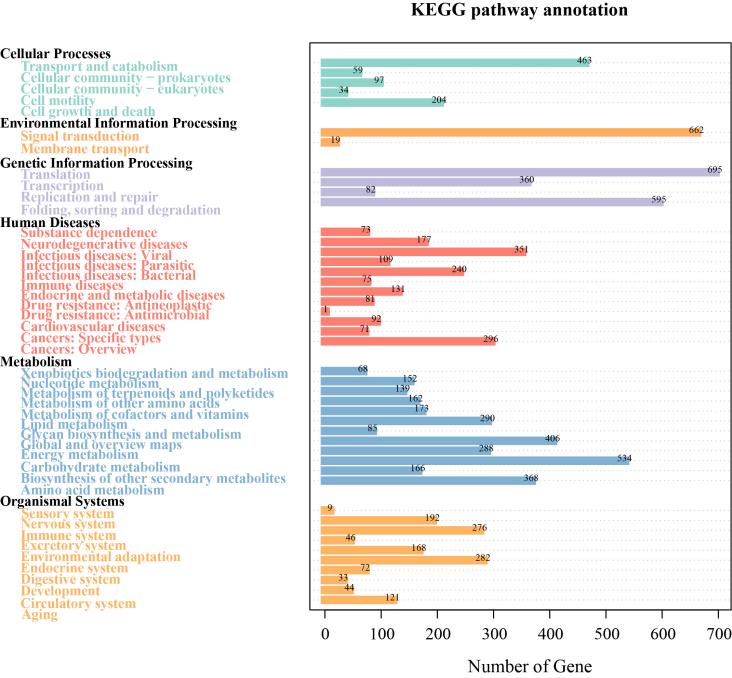


**Supplementary Figure 5.**KEGG pathway classification chart for gene functional annotation.


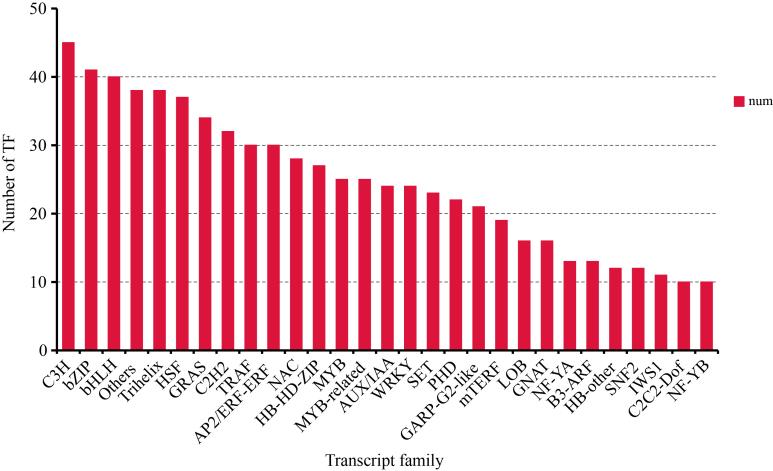


**Supplementary Figure 6.**Transcription Factor Analysis.X-axis: Transcription Factor Families.Y-axis: Number of TFs.


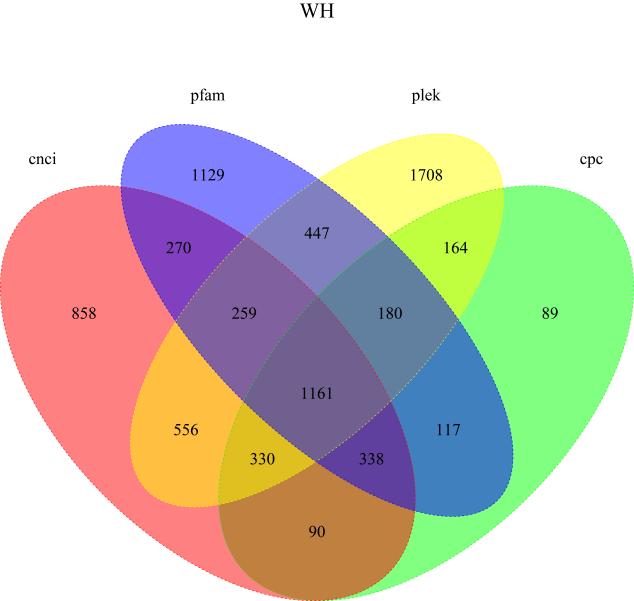


**Supplementary Figure 7.**Venn Diagram of Coding Potential Predictions.The number in each large circle represents the total number of lncRNAs predicted by the respective coding potential prediction software. The overlapping areas indicate the number of lncRNAs shared between the corresponding combinations of tools.
